# Transplanting *Rac1*-silenced bone marrow mesenchymal stem cells promote neurological function recovery in TBI mice

**DOI:** 10.18632/aging.202334

**Published:** 2020-12-19

**Authors:** Dongdong Huang, Felix Siaw-Debrah, Hua Wang, Sheng Ye, Kankai Wang, Ke Wu, Ying Zhang, Hao Wang, Chaojie Yao, Jiayu Chen, Lin Yan, Chun-Li Zhang, Qichuan Zhuge, Jianjing Yang

**Affiliations:** 1 Zhejiang Provincial Key Laboratory of Aging and Neurological Disorder Research, The First Affiliated Hospital of Wenzhou Medical University, Wenzhou 325000, China; 2 Department of Neurosurgery, The First Affiliated Hospital of Wenzhou Medical University, Wenzhou 325000, China; 3 Department of Molecular Biology, University of Texas Southwestern Medical Center, Dallas, Texas 75390, USA

**Keywords:** BMMSCs, NADPH, Rac1, TBI, neurological function recovery

## Abstract

Bone marrow mesenchymal stem cells (BMMSCs)-based therapy has emerged as a promising novel therapy for Traumatic Brain Injury (TBI). However, the therapeutic quantity of viable implanted BMMSCs necessary to initiate efficacy is still undetermined. Increased oxidative stress following TBI, which leads to the activation of nicotinamide adenine dinucleotide phosphate (NADPH) oxidase signaling pathway, has been implicated in accounting for the diminished graft survival and therapeutic effect. To prove this assertion, we silenced the expression of NADPH subunits (p22-phox, p47-phox, and p67-phox) and small GTPase Rac1 in BMMSCs using shRNA. Our results showed that silencing these proteins significantly reduced oxidative stress and cell death/apoptosis, and promoted implanted BMMSCs proliferation after TBI. The most significant result was however seen with *Rac1* silencing, which demonstrated decreased expression of apoptotic proteins, enhanced *in vitro* survival ratio, reduction in TBI lesional volume and significant improvement in neurological function post shRac1-BMMSCs transplantation. Additionally, two RNA-seq hub genes (*VEGFA* and *MMP-2*) were identified to play critical roles in shRac1-mediated cell survival. In summary, we propose that knockdown of *Rac1* gene could significantly boost cell survival and promote the recovery of neurological functions after BMMSCs transplantation in TBI mice.

## INTRODUCTION

Traumatic brain injury (TBI) is a growing public health concern which accounts for over 5.3 million TBI-related disabilities and deaths especially in adolescents and young adults [1, 2]. With an incidence of approximately 1.7 million per year [3], a considerable number of publications have shown that TBI can lead to various neurobehavioral and psychological impairments such as cognitive, sensory, behavioral, psychological stress, etc. [4–6]. The mechanism of brain damage after TBI has been shown to involve both direct mechanical and indirect damage. Indirect damage results from processes such as excitotoxicity, high intracellular calcium levels, oxidative stress, mitochondrial dysfunction, and inflammation [7]. However, despite immense investigative efforts into understanding TBI disease process, there is still the lack of effective treatments to promote recovery of neurological functions, hence prognosis remains unfavorable.

Stem cell transplantation therapy has gained tremendous attention recently. The transplantation of stem cells, such as endogenous neural stem cells (EPS) and induced pluripotent stem cells (iPS), has been considered a potential regenerative medical approach and a promising strategy to treat brain pathologies such as TBI, stroke, and other neurodegenerative diseases [8]. This treatment modality provides the advantage of activating the secretion of trophic factors (e.g., the brain-derived neurotrophic factor (BDNF), glial cell-derived neurotrophic factor (GDNF), and vascular endothelial growth factor (VEGF)), providing anti-inflammatory effects, and activating endogenous neural regeneration-associated pathways [9, 10]. Therefore, by reducing immunogenicity and tumorigenicity, improving accessibility, and resolving pertinent ethical issues, bone marrow mesenchymal stem cells (BMMSCs) transplantation could be considered an appropriate treatment modality for TBI [11, 12]. Unfortunately, owing to the hostile perilesional microenvironment caused by hypoxic-ischemia, oxidative stress, and inflammation post-TBI [13], nearly 99% of transplanted cells fail to thrive [14]. To improve BMMSCs survival rate therefore, some studies have subjected to various degrees of genetic modification [15] and hypoxic pre-conditioning [16]. Nevertheless, there are still some limitations that warrant attention into newer methods to promote BMMSCs survival and curative effect.

The Nox family of nicotinamide adenine dinucleotide phosphate (NADPH) oxidases (Nox1-Nox5 and Duox1-Duox2) are major producers of intracellular reactive oxygen species (ROS), and are involved in chemical modification of molecules, redox reaction, host defense, inflammation, proliferation, and apoptosis [17]. Canonical activation of NADPH oxidases [18] begins with the signaling of Fc or formyl peptide receptors (FPR), which promotes the binding of p47-phox to the transmembrane protein p22-phox [19]. These interactive cascades further precipitate the binding of p67-phox to Nox2 at its N-terminus [20], and subsequently recruit smaller GTPase Rac1 cytoplasmic molecules to also bind to the N-terminus of p67-phox resulting in oxidative stress [21]. In addition to oxidative stress, Rac1 also participates in cytoskeletal dynamics, cell migration and proliferation, apoptosis, transcriptional activation, and DNA damage responses [22, 23]. A previous study has shown that inhibition of ROS-mediated serine-threonine protein kinase (AKT) signaling could significantly reduce implanted BMMSCs population [24]. Thus, we aimed to determine whether inhibiting the expression of NADPH subunits (p22-phox, p47-phox, and p67-phox) and Rac1 could improve survival of BMMSCs at early transplantation stage and promote recovery of neurological functions post TBI.

## RESULTS

### Silencing *Rac1, p22-phox, p47-phox*, or *p67-phox* reduces OGD-induced ROS production

**Figure 1:**
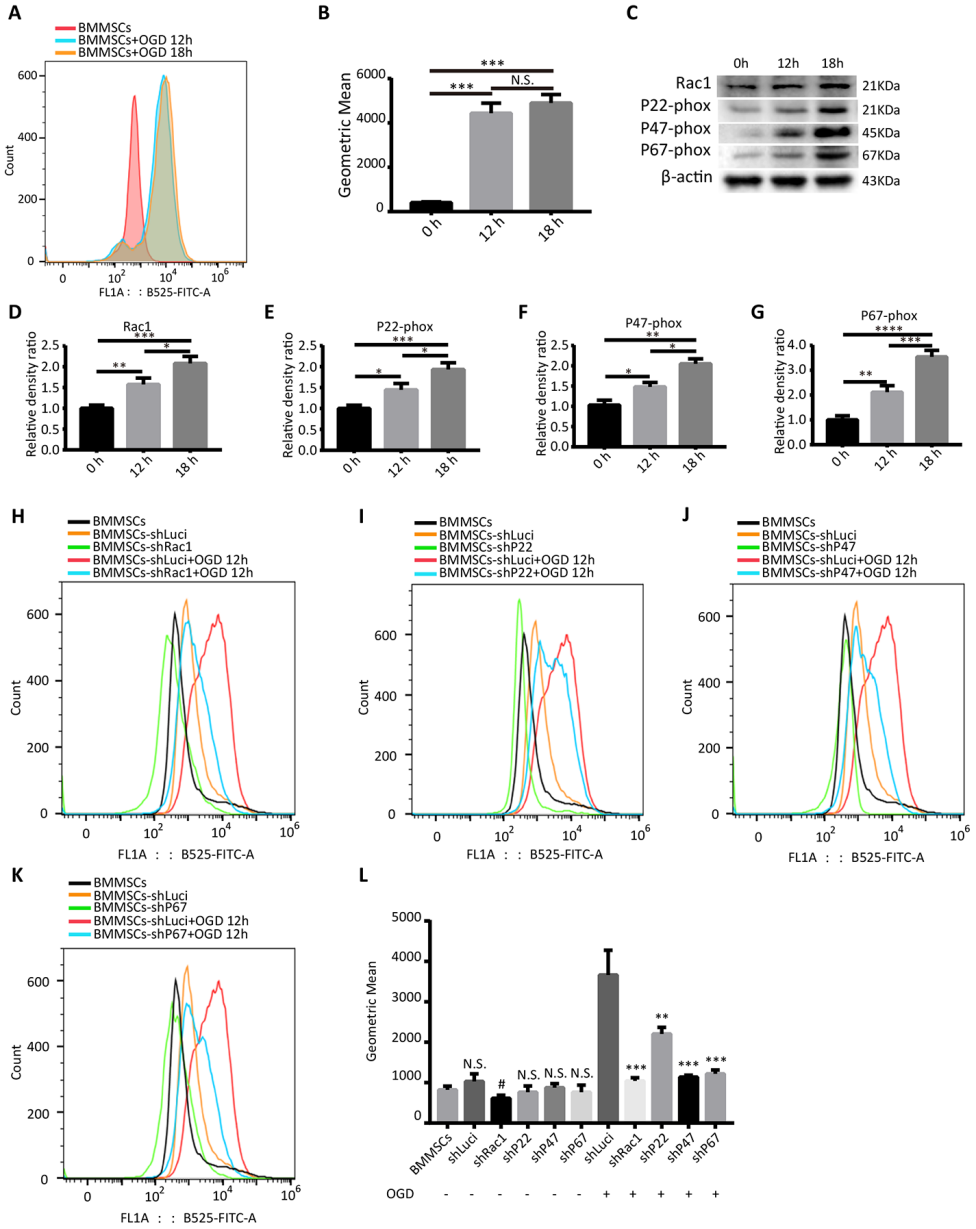
**Silencing *Rac1, p22-phox, p47-phox,* or *p67-pho*x reduces OGD-induced ROS production.** (**A**) Intracellular ROS production, as measured by flow cytometry analysis of DCFH-DA probe fluorescence. (**B**) ROS production after 0 h, 12 h and 18 h OGD treatment. ROS production was notably increased after an OGD. (N.S. no significance, ***P < 0.001, statistically analyzed by one-way ANOVA followed by the Bonferroni correction, n = 3). (**C**–**G**) Rac1, p22-phox, p47-phox, and p67-phox protein expressions in wild type BMMSCs after exposing to different durations of OGD (0 h, 12 h, or 18 h). (*P < 0.05, **P < 0.01, ***P < 0.001, ****P < 0.0001, statistically analyzed by one-way ANOVA followed by the Bonferroni correction, n = 4). (**H**–**K**) Representation of flow cytometric histograms of different transfected BMMSCs and parental BMMSCs with or without an OGD 12 h stimulation. (**L**) Statistical graph showing ROS production of different BMMSCs cell lines under different hypoxia stimulation conditions. In comparison to shLuci, the other lentiviruses showed reduced ROS production to varying degrees. (N.S. no significance versus BMMSCs group, #P < 0.05 versus BMMSCs group, **P < 0.01 versus shLuci+OGD group, ***P < 0.001 versus shLuci+OGD group, statistically analyzed by one-way ANOVA followed by the Bonferroni correction, n = 3). Data are presented as mean ± SD.

Using 2’, 7’-dichlorodihydrofluorescein diacetate (DCFH-DA) probe and flow cytometry, we first demonstrated that oxygen-glucose deprivation (OGD) significantly increased intracellular ROS levels in BMMSCs (Figure 1A, 1B). However, there was no statistically significant difference between the OGD 12 h and OGD 18 h treatment groups (Figure 1A, 1B). In addition, western blot analyses showed that OGD treatment not only induced the expression of NADPH subunits (p22-phox, p47-phox, and p67-phox; Figure 1C, 1E–1G) but also Rac1 (Figure 1C, 1D), suggesting that OGD could activate the NADPH oxidase signaling pathway.

Subsequently, *Rac1, p22-phox, p47-phox,* and *p67-phox* expressions in BMMSCs were silenced by a lentiviral-mediated transfection of the shRNA lentiviruses. Analysis of mCherry expression confirmed that the infection efficiency was almost 80%, after 3 days of transfection (Supplementary Figure 1). In order to confirm the knockdown efficiencies of shRac1, shP22-phox, shP47-phox and shP67-phox, transfected BMMSCs were treated with OGD 12 h, and analyzed with western blot and quantitative reverse transcription-polymerase chain reaction (qRT-PCR). Results showed that, in comparison to the BMMSCs-shLuci (cells transfected with a luciferase-silencing lentivirus), shRac1 decreased *Rac1* expression by approximately 75%, whereas other lentiviruses silenced their respective genes by about 50% (Supplementary Figure 2).

To determine whether silencing of these genes in BMMSCs could reduce OGD-induced ROS production, flow cytometry was used to detect the intracellular ROS levels. As expected, transfecting cells with the different lentiviruses (shRac1, shP22-phox, shP47-phox and shP67-phox) significantly reduced intracellular ROS formation to varying degrees after OGD (Figure 1H–1K). Meanwhile, comparing the ROS production in parental BMMSCs to that of transfected BMMSCs with no OGD stimulation, showed no statistical difference between them except for the shRac1 group (Figure 1L). Silencing *Rac1* was seen to have the most considerable effect on ROS production and therefore knocking down NADPH subunits and Rac1 could significantly inhibit OGD-induced ROS production.

### Silencing *Rac1, p22-phox, p47-phox,* or *p67-phox* promotes BMMSCs proliferation and viability after an OGD treatment

**Figure 2:**
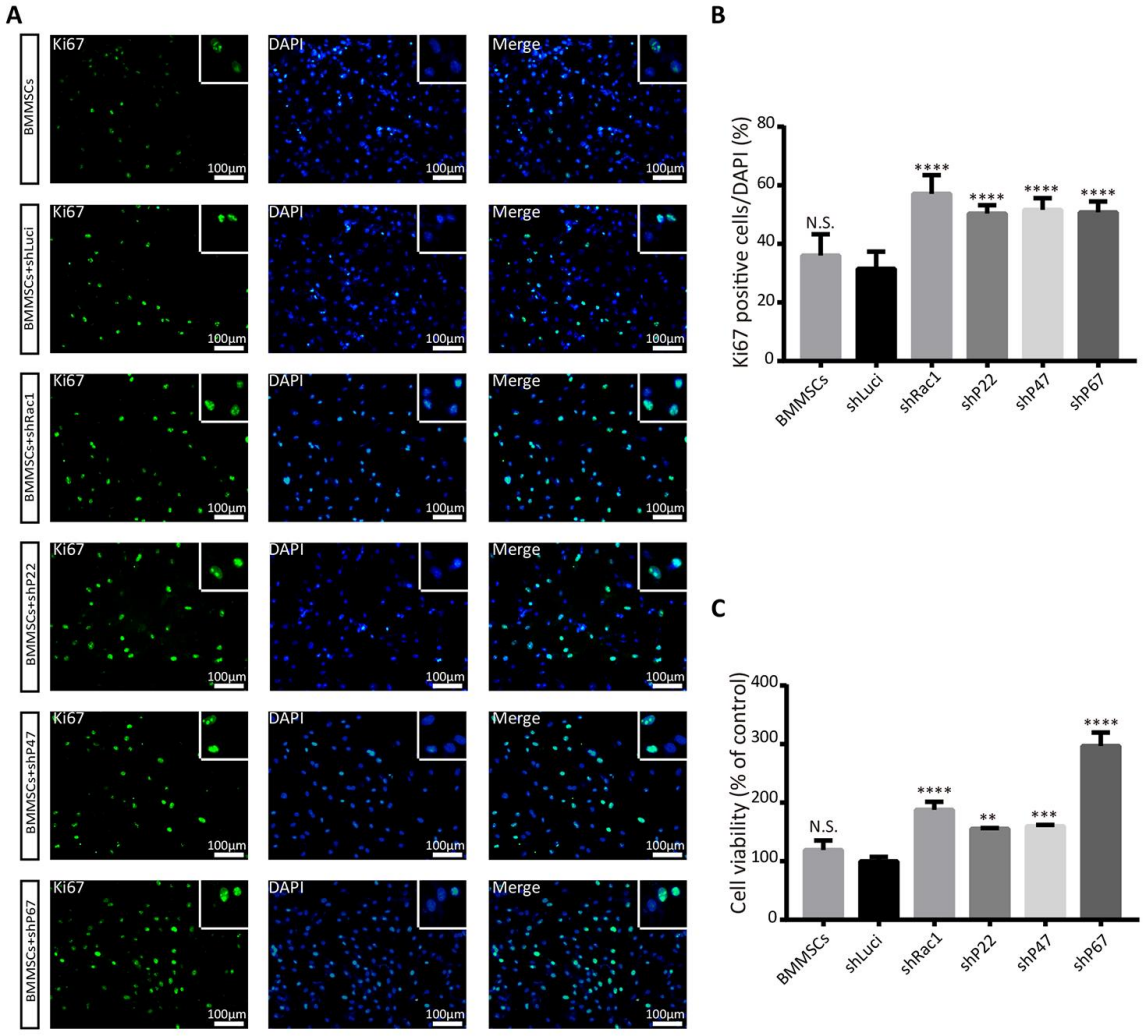
**Silencing *Rac1, p22-phox, p47-phox,* or *p67-phox* promotes BMMSCs proliferation and viability after an OGD treatment.** (**A**) Images representing Ki67 (green) expression in modified BMMSCs (transfected with shRac1, shP22, shP47, shP67, shLuci) and wild type BMMSCs, and counterstained with DAPI (blue). In comparison with the shLuci-transfected cells, other BMMSCs cell lines showed more Ki67(+) cells. The upper right corner insert shows a zoomed-in image of the local area with scale bar of 100μm. (**B**) The Ki67(+)/DAPI ratio was higher for BMMSCs transfected with shRac1, shP22, shP47, or shP67. (N.S. no significance, ****P < 0.0001, statistically analyzed by one-way ANOVA followed by the Bonferroni correction, n = 4). (**C**) BMMSCs viability, as measured by the CCK-8 assay, showed improved BMMSCs survival after transfection with shRac1, shP22, shP47, or shP67 lentivirus. (N.S. no significance, **P < 0.01, ***P < 0.001, ****P < 0.0001, statistically analyzed by one-way ANOVA followed by the Bonferroni comparison, n = 3). Data are presented as mean ± SD.

Having established that silencing NADPH subunits and Rac1 inhibit OGD-induced ROS production, we sort to understand whether this phenomenon promotes BMMSCs viability and proliferation. Recent studies have confirmed that oxidative stress can trigger cellular apoptosis by inducing DNA damage, reducing the duration of pre-metaphase [25, 26], and eventually influencing cell proliferation and viability. To determine whether silencing NADPH signaling could rescue BMMSCs from an OGD-induced cell death/apoptosis, Ki67 and cell counting kit-8 (CCK-8) assays were used to investigate cell proliferation and viability. From our Ki67(+) positive results, there was no significant difference between the parental BMMSCs and luciferase BMMSCs group. In contrast, BMMSCs with knocked-down NADPH subunits and Rac1 had significantly more Ki67(+) cells compared to the luciferase lentiviruses transfected BMMSCs (Figure 2A, 2B). To further confirm earlier results, cell viability assay with CCK-8 assay was performed after OGD. Consistent with the results of the Ki67 staining, cell growth was significantly enhanced in *Rac1, p22-phox, p47-phox,* or *p67-phox* knocked down cells (Figure 2C), with BMMSCs-shRac1 and BMMSCs-shP67 cells showing the fastest-growth. Similar results were also obtained from the trypan blue cell viability assays (Supplementary Figure 3A–3F). In summary, we showed that silencing *Rac1, p22-phox, p47-phox,* and *p67-phox* in BMMSCs enhanced cell proliferation and viability after OGD, with shRac1 and shP67 having the most considerable effects.

### shRac1 and shP67 transfection reduce cell death/apoptosis

**Figure 3:**
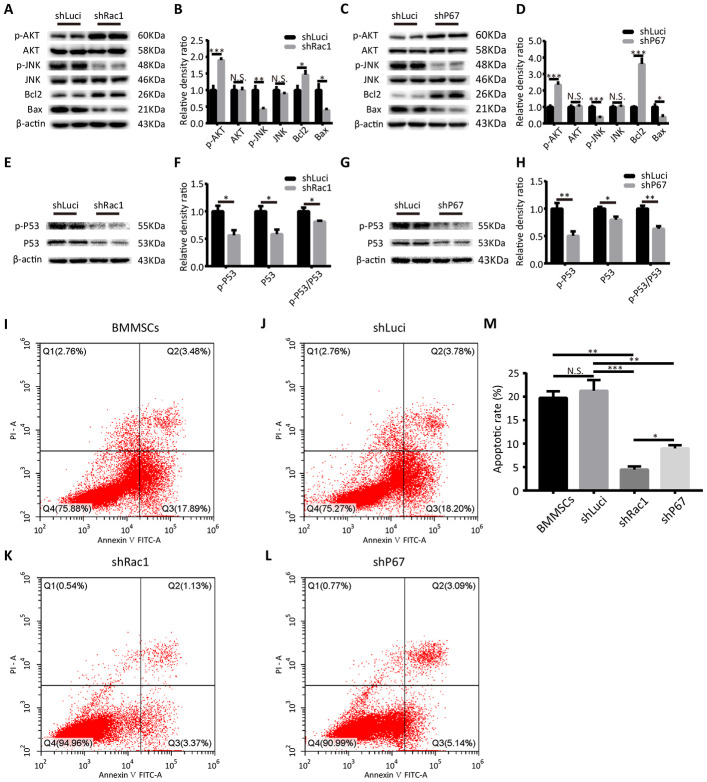
**shRac1 and shP67 transfection reduce cell death/apoptosis.** (**A**, **B**) Quantification of pro-apoptotic (p-JNK, JNK, Bax) and anti-apoptotic (p-AKT, AKT, Bcl-2) protein expressions in shRac1 and shLuci BMMSCs post-OGD 12 h. β-actin was used as the housekeeping protein. (N.S. no significance, *P < 0.05, **P < 0.01, ***P < 0.001, statistically analyzed by the Student’s t-test, n = 4). (**C**, **D**) Pro-apoptotic (p-JNK, JNK, and Bax) and anti-apoptotic (p-AKT, AKT, and Bcl-2) protein expressions in shP67 and shLuci BMMSCs post-OGD 12 h, β-actin was used as the housekeeping protein. (N.S. no significance, *P < 0.05, ***P < 0.001, statistically analyzed by the Student’s t-test, n = 4). (**E**–**H**) p53 expression and activation as reflected by total and phospho-p53 protein expressions. In shRac1- and shP67-transfected BMMSCs post-OGD treatment, p53 expression and activity were found to be decreased. (N.S. no significance, *P < 0.05, **P < 0.01, statistically analyzed by the Student’s t-test, n = 4). (**I**–**M**) Flow cytometry measured apoptosis of shLuci, shRac1, shP67 BMMSCs and wild type BMMSCs. In comparison with shLuci, shP67 and wild type BMMSCs, shRac1 BMMSCs showed better anti-apoptotic ability. (N.S. no significance, *P < 0.05, **P < 0.01, ***P < 0.001, statistically analyzed by one-way ANOVA followed by the Bonferroni correction, n = 3). Data are presented as mean ± SD.

Multiple studies have shown that ROS is a crucial upstream inducer of cell death/apoptosis activation of the p53, phosphoinositide 3-kinase (PI3K)/AKT, c-Jun N-terminal Kinase (JNK), and p38 signaling pathways [27, 28]. For example, Santabárbara-Ruiz et al. reported that JNK signaling selectively promoted apoptosis and modulated proliferation, which was related to a shift in the ROS production [29]. Therefore, using western blot we investigated the expressions of several proteins associated with ROS-mediated cell death/apoptosis in shRac1 and shP67-BMMSCs. As a serine and threonine protein kinase, AKT can inhibit the conformational changes of Bax and down-regulate the nuclear expression of the tumor suppressor protein p53 hence inhibit apoptosis [30]. We found that phosphorylation and activation of AKT, was markedly increased in the shRac1 and shP67 BMMSCs in comparison to that of shLuci cells. However, there was no significant difference when the total AKT protein expression was quantified (Figure 3A–3D). In contrast, the expression of phosphorylated JNK decreased with gene silencing, whereas total JNK expression did not change (Figure 3A–3D). Additionally, we investigated the expressions of apoptotic proteins Bax and Bcl-2, which are downstream mediators of the JNK signaling. As expected, we found that the expression of anti-apoptotic Bcl-2 was increased, whereas that of pro-apoptotic Bax had decreased in shRac1 and shP67 BMMSCs, which were consistent with downstream mediators of AKT and JNK (Figure 3A–3D). From a previous study, ROS was discovered to directly cause DNA damage and initiate p53 mediated apoptosis [31]. Thus, we further investigated the total and the phosphorylated p53 expressions, and evaluated the phospho-/total-p53 ratio. In contrast to the shLuci control cells, the proportion of shRac1 and shP67 BMMSCs with phospho-p53 were significantly reduced (Figure 3E–3H). To further verify the anti-apoptotic effect, apoptotic cell rate was determined using flow cytometry analyses (Figure 3I–3L). Compared with parental BMMSCs and shLuci cell lines, the apoptotic rates of shRac1 and shP67 were significantly decreased, with shRac1's group showing the most significant decrease (Figure 3M). Altogether, our results suggested that BMMSCs transfected with shRac1 or shP67 lentiviruses inhibited apoptosis in these cells.

### Transplanting BMMSCs-shRac1 promotes recovery of the neurological functions and reduces brain edema after TBI

**Figure 4:**
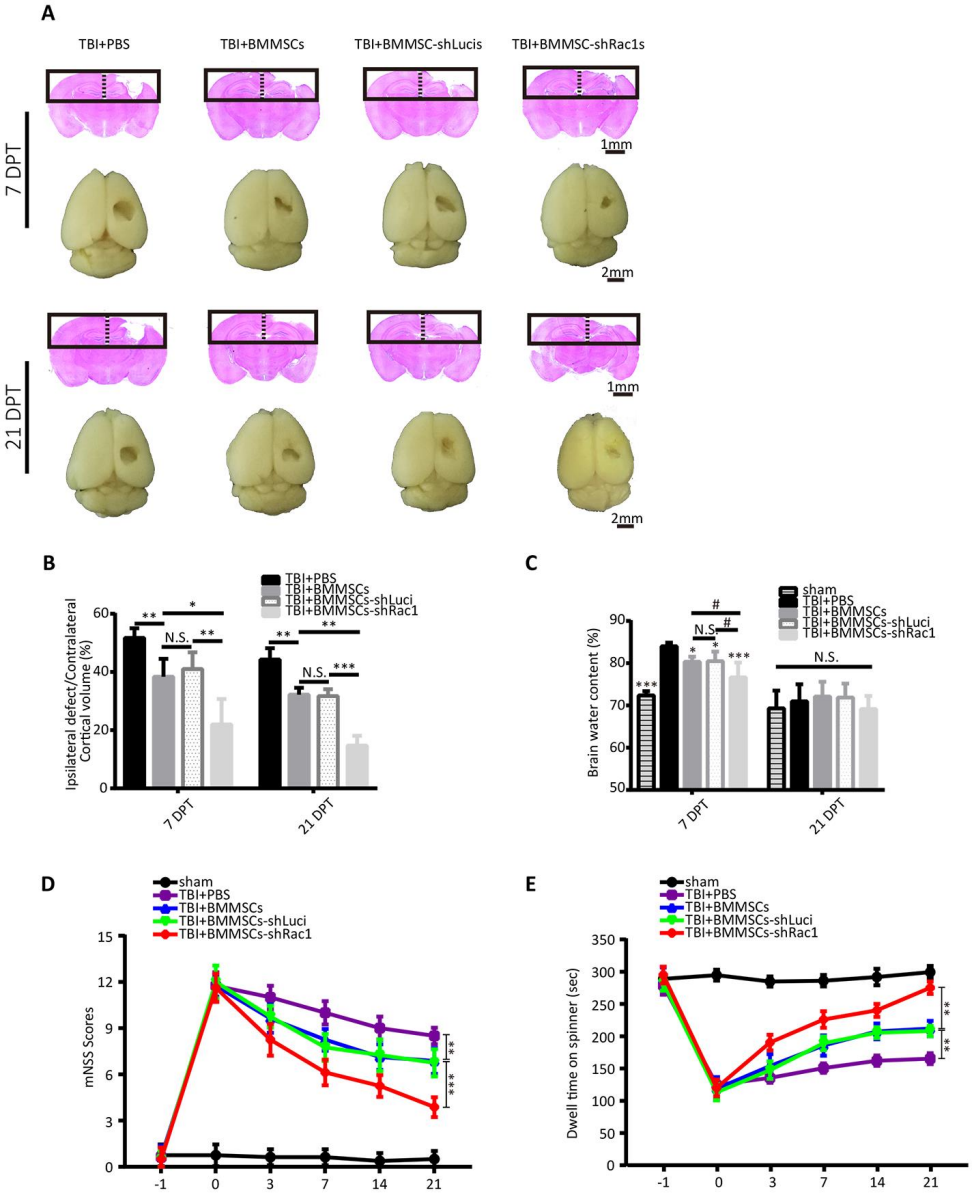
**Transplanting BMMSCs-shRac1 promotes the recovery of neurological functions and reduces brain edema after TBI.** (**A**) Images displaying the whole brain and ipsilateral TBI brain stained with H&E 7 days and 21 days after BMMSCs (parental BMMSCs, BMMSCs-shLuci, BMMSCs-shRac1) transplantation or after PBS injection. The scale bar of 1 mm and 2 mm is indicated, respectively. (**B**) Quantification of lesional volume in the BMMSCs-transplanted versus placebo (PBS) mice. The lesional size was smallest in BMMSCs-shRac1 transplanted mice at 7 days post transplantation (DPT) and 21 DPT. (N.S. no significance, *P < 0.05, **P < 0.01, ***P < 0.001, statistically analyzed by one-way ANOVA followed by the Bonferroni correction, n = 5). (**C**) Quantitative analysis of brain moisture content in each group. Mice treated with parental BMMSCs, BMMSCs-shLuci, and BMMSCs-shRac1 had less edema compared with PBS injection group at 7 DPT, with the BMMSCs-shRac1 group showing minimal edema. No significance was found at 21 DPT. (N.S. no significance, #P < 0.05, *P < 0.05, ***P < 0.001, statistically analyzed by one-way ANOVA followed by the Bonferroni correction, n = 6). (**D**) mNSS scores for mice treated with PBS, parental BMMSCs, BMMSCs-shLuci, or BMMSCs-shRac1, with the latter achieving the lowest score. (**P < 0.01, ***P < 0.001, statistically analyzed by two-way ANOVA followed by the Bonferroni correction, n = 8). (**E**) Rotarod test of mice treated with PBS, parental BMMSCs, BMMSCs-shLuci, or BMMSCs-shRac1, where the latter achieved the maximum stay-on time at each time point after transplantation (**P < 0.01, statistically analyzed by two-way ANOVA followed by Bonferroni correction, n = 8). Data are presented as mean ± SD.

Based on our previous results, BMMSCs-shRac1 was selected as our lead cell line to further investigate whether it could reduce TBI lesional volume and promote recovery of neurological functions in mice TBI model. TBI lesional volume was measured at 7 and 21 days after BMMSCs transplantation (Figure 4A). Results showed decreased parenchymal lesional size for all BMMSCs-treated mice compared to PBS treatment group, with the BMMSCs-shRac1 transplanted group showing the most significant decrease (Figure 4B). With regards to investigating the effect on brain edema, our results showed that BMMSCs transplantation significantly improved edema at 7 days post transplantation (DPT), with significant effect seen in BMMSCs-shRac1 group (Figure 4C). At 21 days however, no significant difference between these groups (Figure 4C). To assess the extent of neurological function recovery, the modified neurological severity score (mNSS) and the rotarod test were used in a blinded manner. From day 3 to day 21 post-transplantation, both tests showed significant neurological functions recovery in TBI+BMMSCs-shRac1 group compared to the TBI+BMMSCs-shLuci mice (Figure 4D, 4E). In contrast, there was no major neurobehavioral and histological distinction between the TBI+BMMSCs and TBI+BMMSCs-shLuci groups (Figure 4B–4E). Furthermore, our data showed that the TBI+BMMSCs-shRac1 group exhibited significant improvement in neurological functions compared to the BMMSCs wild type group.

### shRac1 improves neuron survival by promoting BMMSCs survival after transplantation into TBI mice

**Figure 5:**
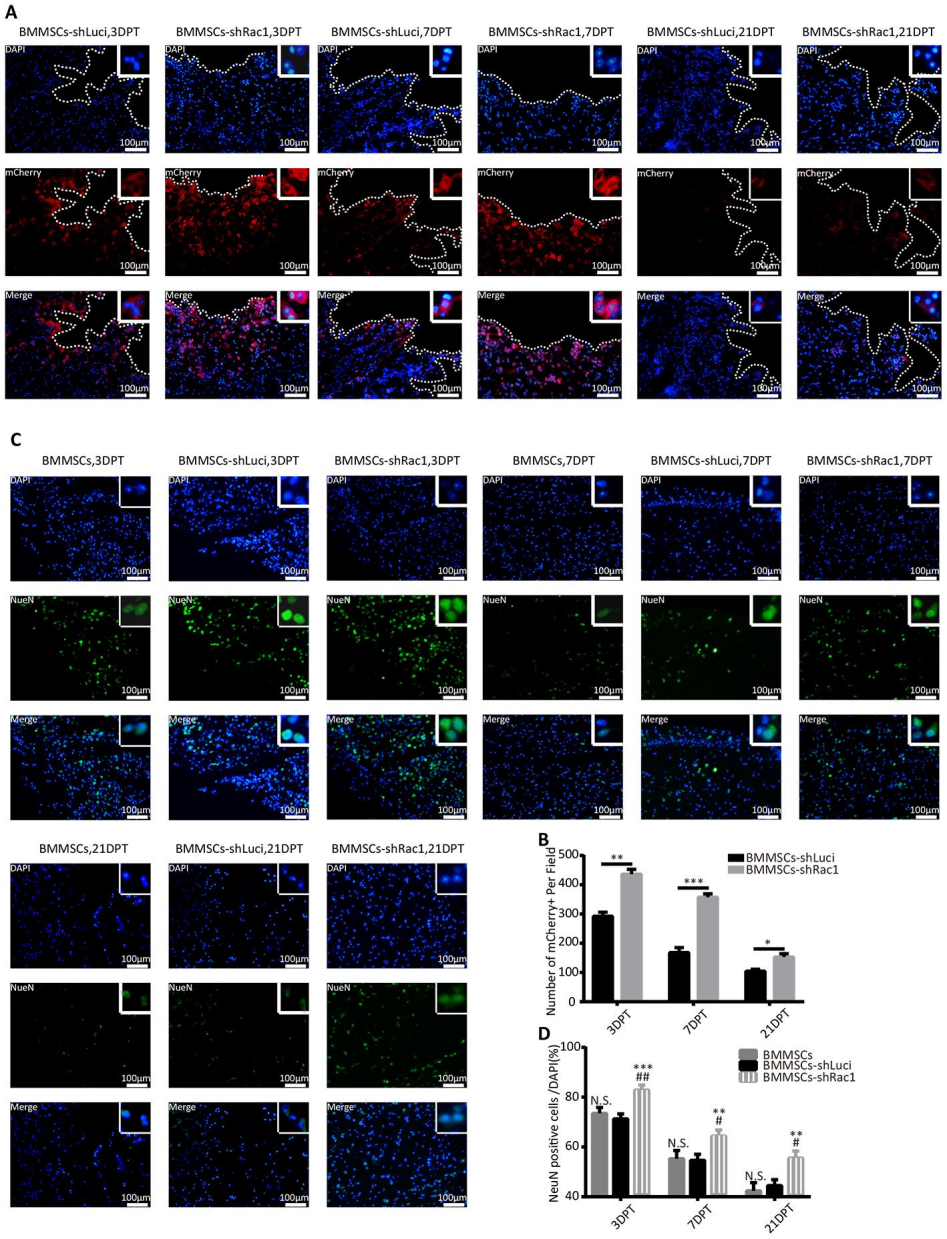
**shRac1 improves neuron survival by promoting BMMSCs transplant survival after transplantation into TBI mice.** (**A**) Images representing surviving BMMSCs-shLuci and BMMSCs-shRac1 at 3, 7 and 21 DPT. Surviving BMMSCs in the lesional site were labeled with mCherry (red) and counterstained with DAPI (blue). Lesional boundaries were outlined by a continuous imaginary line. The upper right corner insert shows a zoomed-in image of the local area with a scale bar of 100μm. (**B**) mCherry (+) cell counts per field showed more BMMSCs-shRac1 cells survived than the BMMSCs-shLuci cells at day 3, day 7 and day 21 (*P < 0.05, **P < 0.01, ***P < 0.001, statistically analyzed by the Student’s t-test, n = 6). (**C**) Representative images of the surviving neurons at 3, 7 and 21 DPT, neuron surviving in the lesion location were labeled with NeuN (green) and counterstained with DAPI (blue). The upper right corner insert shows a zoomed-in image of the local area with scale bar of 100 μm. (**D**) NeuN (+)/DAPI ratio was higher for mice transplanted with BMMSCs-shRac1 than those transplanted with parental BMMSCs and BMMSCs-shLuci at different time points. (N.S. no significance, **P < 0.01, ***P < 0.001, #P < 0.05 versus parental BMMSCs transplanted group, statistically analyzed by Student’s t-test, n = 6). Data are presented as mean ± SD.

To investigate factors that enable transplanted BMMSCs-shRac1 to significantly improve cognitive function as compared to BMMSCs-shLuci, immunofluorescence tissue staining was done to track the survival of transplanted cells and neurons at 3, 7 and 21- days post-treatment. We verified the presence of grafted cells at lesion site by the expression of mCherry reporter protein in transfected cells (Figure 5A). Our results showed that in comparison with BMMSCs-shLuci transplantation, BMMSCs survival at 3, 7 and 21-days post-transplantation were significantly improved by shRac1 transfecting (Figure 5B). Grafted cells were seen distributed throughout the cerebral hemisphere, and more especially at lesional site but gradually diminished from 3 to 21 days (Figure 5B). We also observed a significant reduction in NeuN expressing neurons in BMMSCs-shLuci transplanted group as compared to BMMSCs-shRac1 transplanted group (Figure 5C, 5D). As compared to parental BMMSCs transplanted group, tremendous neurons survival was seen in BMMSCs-shRac1 transplanted group (Figure 5D). Altogether, these results suggest that shRac1-mediated improvement in neurons and BMMSCs survival and hence improvement in neurological functional recovery.

### Identifying changes in gene expression in the shRac1-modified BMMSCs after an OGD treatment

**Figure 6:**
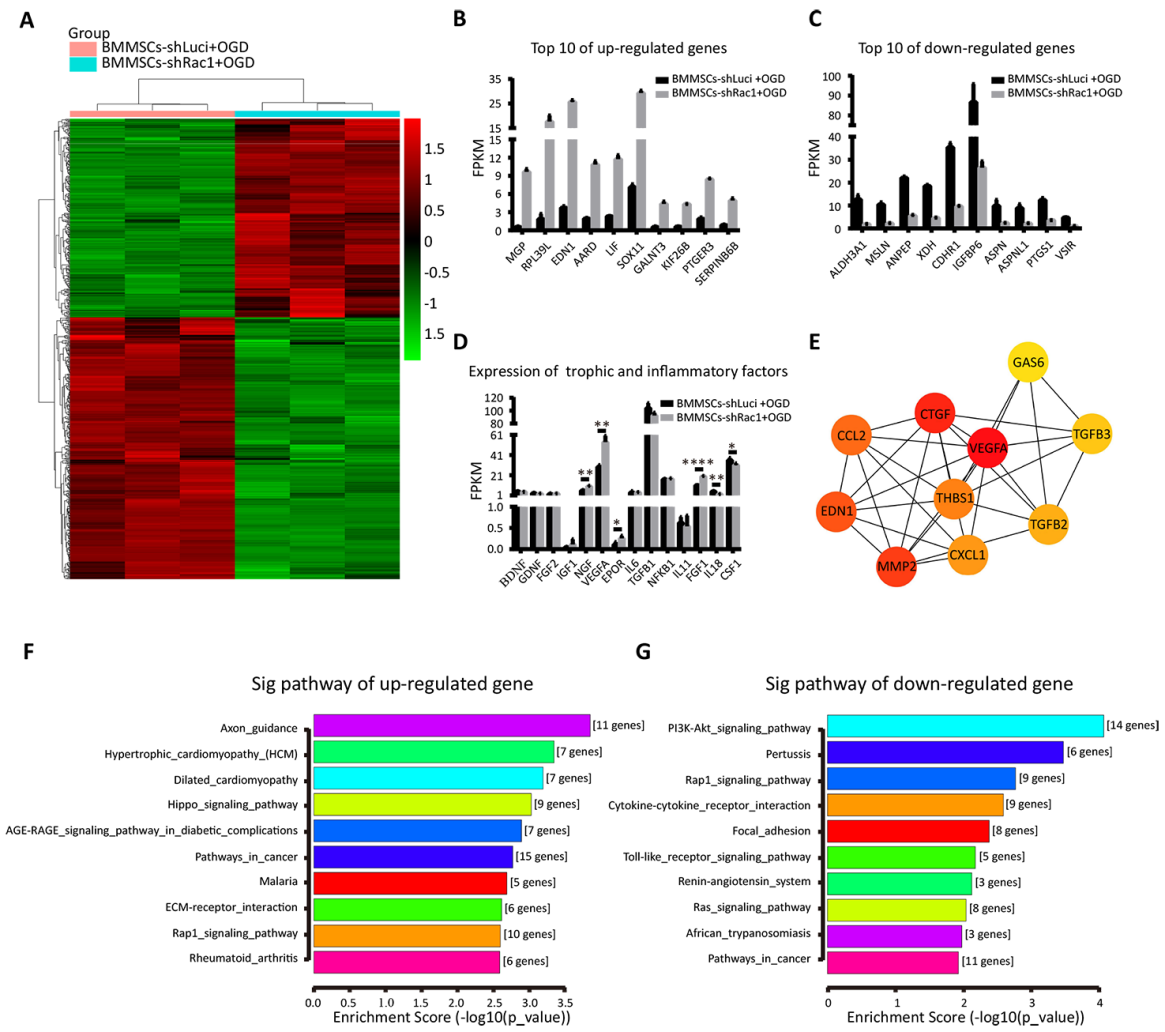
**Identifying changes in gene expression in the shRac1-modified BMMSCs after an OGD treatment.** (**A**) The relative gene expressions in BMMSCs-shLuci+OGD and BMMSCs-shRac1+OGD samples. The ordinate represents genes, and the abscissa indicates samples classification. Red color indicates higher expression, and green color indicates lower expression. (**B**, **C**) The top 10 up-regulated and down-regulated genes when comparing BMMSCs-shRac1+OGD with BMMSCs-shLuci+OGD. (**D**) The expressions of trophic (NGF, VEGFA, EPOR, and FGF-1) were significantly increased but with inflammatory factors (IL-18 and CSF-1) decreasing significantly in BMMSCs-shRac1 cells. (**E**) Hub gene network. (**F**, **G**) The KEGG enrichment pathway analysis of BMMSCs-shLuci+OGD and BMMSCs-shRac1+OGD RNA-Seq data, enrichment scores ranked from the highest to the lowest. Data are presented as mean ± SD.

To understand the potential mechanism of promoting cell survival and post-TBI functional recovery by BMMSCs-shRac1, we conducted RNA-seq and bioinformatics analyses (heatmap, Gene Ontology (GO), KEGG (Kyoto Encyclopedia of Genes and Genomes), and protein-gene interaction network) of the different BMMSCs lines. Firstly, we investigated the effect of gene knockdown and/or OGD stimulation on BMMSCs-related markers to determine how they alter BMMSCs characteristics [32]. Results showed stable BMMSCs markers generally, although some changes were observed in CD90 and CD29 expression (Supplementary Figure 5). Secondly, we compared parental BMMSCs and BMMSCs-shLuci cells to investigate whether the transfection process resulted in any changes in gene expression. While KEGG analyses (Supplementary Figure 4B, 4C) showed respective up and down regulation of major genes in the tumor necrosis factor (*TNF*) and glutamatergic signaling pathways respectively, the predominant changes observed were those associated with negative regulation of cellular processes and responses to oxygen levels (Supplementary Figure 4D, 4E). More importantly, we compared the differences in gene expression between BMMSCs-shLuci+OGD and BMMSCs-shRac1+OGD in a heatmap (Figure 6A). As shown in Figure 6F, 6G, the following KEGG pathways were enriched when comparing BMMSCs-shRac1+OGD and BMMSCs-shLuci+OGD datasets i.e. up-regulated pathway were the axon guidance, hypertrophic cardiomyopathy (HCM), and dilated cardiomyopathy whiles down-regulated pathways were the PI3K-AKT signaling pathway, Pertussis, and Rap1 signaling pathway. The top 10 up-regulated and down-regulated genes with the most notable fold-change have been presented in a histogram (Figure 6B, 6C). Moreover, using the STRING database, we established a protein interaction network for 317 detected up-regulate genes (Supplementary Table 3) in BMMSCs-shLuci+OGD and BMMSCs-shRac1+OGD treatment groups. Finally, the Cytoscape software was utilized to identify the top 10 hub genes: vascular endothelial growth factor A (*VEGFA*), connective tissue growth factor (*CTGF*), matrix metalloproteinase 2 (*MMP-2*), endothelin 1 (*EDN1*), chemokine (C-C motif) ligand 2 (*CCL2*), thrombospondin 1 (*THBS1*), chemokine (C-X-C motif) ligand 1 (*CXCL1*), transforming growth factor beta (*TGFβ*)-2, *TGFβ-3*, and growth arrest specific 6 (*GAS6*; Figure 6E). To better understand the mechanism by which shRac1 modified BMMSCs to improve the prognosis of TBI mice, we compared the expressions of several trophic and inflammatory factors, which were considered as possible promoters of TBI [8]. Notably, our results showed that in comparison with the BMMSCs-shLuci cells, BMMSCs-shRac1 cells had enhanced expressions of trophic factors (the nerve growth factor (*NGF*), *VEGFA*, erythropoietin receptor (*EPOR*), and fibroblast growth factor 1 (*FGF-1*)) and reduced expressions of inflammatory factor (interleukin 18 (*IL-18*) and colony-stimulating factor 1 (*CSF-1*)) (Figure 6D).

### shVEGFA and shMMP-2 attenuate the shRac1-mediated effects in BMMSCs

**Figure 7:**
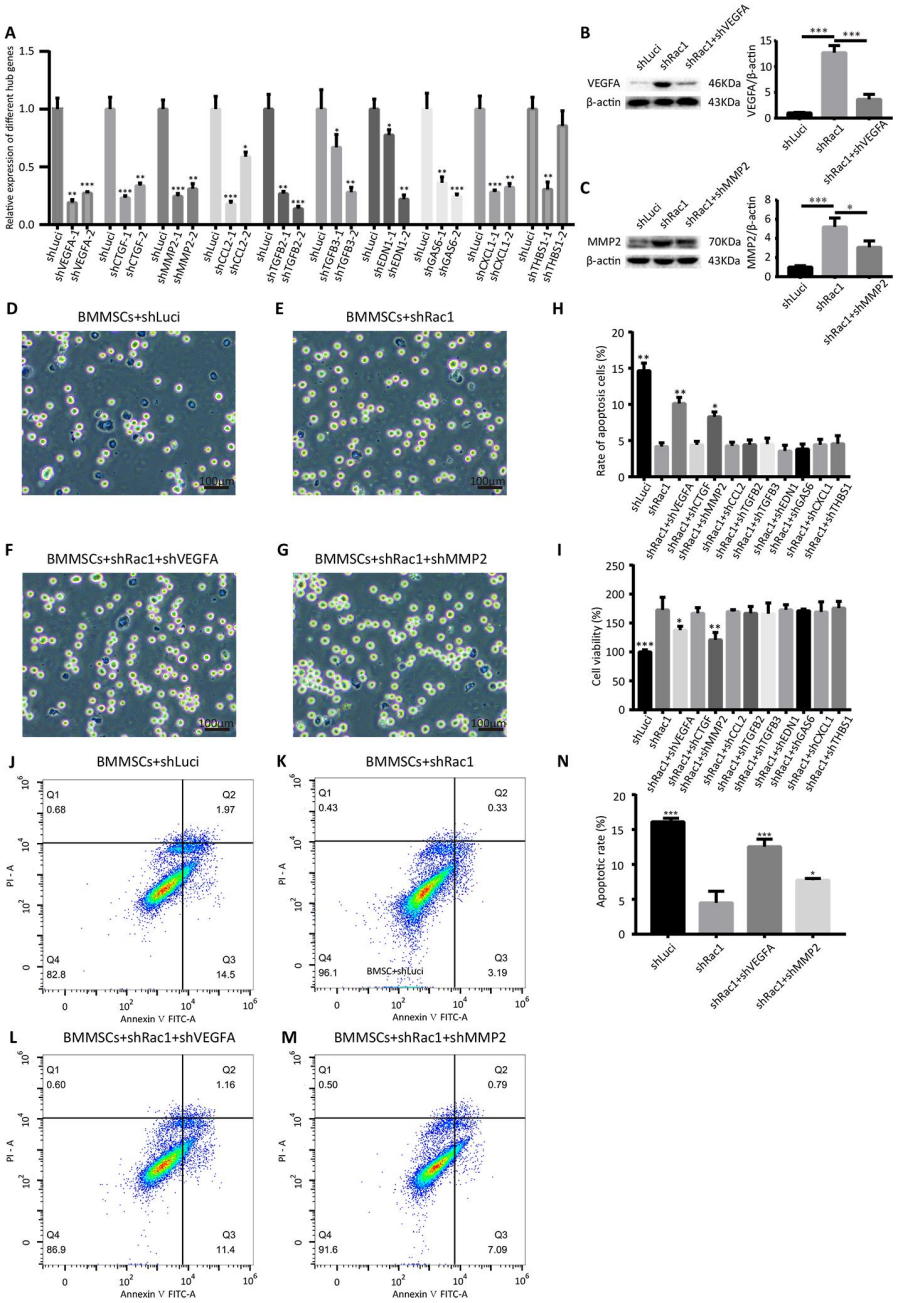
**shVEGFA and shMMP-2 attenuate the shRac1-mediated effects in BMMSCs.** (**A**) qRT-PCR analysis confirmed shRNA knockdown of each hub gene. (N.S. no significance, *P < 0.05, **P < 0.01, ***P < 0.001, statistically analyzed by one-way ANOVA followed by the Bonferroni correction, n = 3). (**B**, **C**) VEGFA and MMP2 protein expression in shLuci, shRac1, shRac1-shVEGFA and/or shRac1-shMMP2 BMMSCs post-OGD 12 h, β-actin was used as the housekeeping protein. (*P < 0.05, ***P < 0.001, statistically analyzed by one-way ANOVA followed by the Bonferroni correction, n = 3). (**D**–**G**) Images showing trypan blue-stained shLuci, shRac1, shRac1+shVEGFA, and shRac1+shMMP-2 cells captured under a bright field microscope with scale bar of 100 μm. (**H**) Quantitative analyses of trypan blue staining showing increased apoptotic rate of BMMSCs-shRac1 co-transfected with shVEGFA or shMMP-2 lentiviruses in comparison with the BMMSCs transfected with shRac1 alone. (N.S. no significance, *P < 0.05, **P < 0.01, statistically analyzed by one-way ANOVA followed by the Bonferroni correction, n = 3). (**I**) BMMSCs-shRac1 survival was attenuated by the co-transfection of shVEGFA and shMMP-2 lentiviruses, but not by other shRNA, as measured by the CCK-8 analysis (N.S. no significance, *P < 0.05, **P < 0.01, ***P < 0.001, statistically analyzed by one-way ANOVA followed by the Bonferroni correction, n = 3). (**J**–**N**) Flow cytometry measured apoptosis of different BMMSCs cell lines. Data further show anti-apoptosis effect of BMMSCs-shRac1 can be weakened by shVEGFA or shMMP-2 co-transfection. (*P < 0.05, ***P < 0.001, statistically analyzed by one-way ANOVA followed by the Bonferroni correction, n = 3). Data are presented as mean ± SD.

From the 10 hub genes that we identified, we hypothesized that at least one of them played a critical role in improving BMMSCs survival. Two shRNAs were constructed for each hub gene, and the knockdown efficiency was confirmed using qRT-PCR (Figure 7A). CCK-8, trypan blue staining assay and Annexin V-FITC/propidium iodide apoptosis assays were also used to investigate the potential roles of these 10 hub genes in shRac1-mediated cell survival. Our results showed that, in comparison with the post OGD-BMMSCs transfected with shRac1 lentivirus alone, the BMMSCs co-transfected with either shRac1 and shVEGFA or shRac1 and shMMP-2 group exhibited more apoptosis, as shown by an increased trypan blue staining (Figure 7D-7H), and lower cell viability, as shown by the CCK-8 assay (Figure 7I). In contrast however, there were no significant changes to these parameters when shRac1 was co-transfected with other shRNA lentiviruses. Furthermore, as compared with shRac1 transfected alone (4.49%±1.67%), the apoptotic rate of co-transfected shRac1-shVEGFA (12.54%±1.10%) and co-transfected shRac1-shMMP-2 (7.73%±0.25%) were significantly increased (Figure 7J–7N). In addition, the apoptotic result conducted by flow cytometry was similar to trypan blue staining analysis. The expressions of *VEGFA* or *MMP2* in different groups (BMMSCs-shLuci, BMMSCs-shRac1, BMMSCs-shRac1-shVEGFA and/or BMMSCs-shRac1-shMMP2) showed upregulation of *VEGFA* and *MMP2* in BMMSCs-shRac1 group, but partial inhibition or downregulation in BMMSCs-shRac1-shVEGFA and BMMSCs-shRac1-shMMP2 groups (Figure 7B, 7C). To sum up, our results support the hypothesis that shRac1-mediated enhancement of BMMSCs survival can be partially reversed by inhibiting the expressions of these two hub genes.

## DISCUSSION

TBI has been a vexing and an intractable global health problem, with high mortality rates and substantial burden to individual, family, and healthcare system [33]. The pathophysiological mechanism of TBI is complex which involves both primary diffuse axonal injury and secondary injury. Secondary injury which is caused by excessive releases of excitatory amino acids and neurotransmitters, an imbalance in the levels of calcium and potassium, hypoxia, mitochondrial dysfunction, oxidative stress, increase in the membrane permeability, and inflammation causes what is known as indirect injury [1, 7]. In addition to the traditional surgical intervention and neurotrophic treatments modalities [34], BMMSCs transplantation has received tremendous attention as a promising new therapeutic strategy for TBI [35]. However, the effectiveness of this treatment modality is somewhat highly dependent on the pathophysiological processes around the perilesional microenvironments [36]. As such considerable amount of effort has been made to precondition BMMSCs for prospective environment in an attempt to improve the efficacy of BMMSCs grafts. These strategies include genetic modification of BMMSCs, synergistic treatment with chemotherapeutic agents, hypoxic preconditioning etc., for which efficacy has been reported [8]. For example, Wu et al. [15] enhanced the survival of BMMSCs via neurotrophin 3 (*NT3)P75-2* gene modification, while Hu et al. [37] markedly improved the survival of BMMSCs by preconditioning with a calpain inhibitor (MDL28170). Although these known modifications improve cell survival, newer strategies to boost transplanted BMMSCs survival to therapeutic efficacy continues to be an urgent need. In this study, we sought to enhance survival of transplanted BMMSCs and improve recovery of neurological function after TBI by inhibiting NADPH subunits (p22-phox, p47-phox, and p67-phox) and Rac1 expressions. From our results, knockdown of NADPH-related genes in BMMSCs grafts reduces ROS production and apoptosis, favors graft survival, and improves post-transplantation TBI-prognosis.

Since NADPH subunits (p22-phox, p47-phox, and p67-phox) and Rac1 have been studied to contribute to ROS production, the knockdown of Rac1 with shRac1 is expected to produce significant inhibition of oxidative stress. As demonstrated by our results Rac1-silenced BMMSCs in comparison to other groups, had obvious advantages as quantified by experimental determination of ROS production, Ki67 staining, trypan blue staining and/or flow cytometry detection of apoptosis. Nox2 activation, the main mechanism of ROS formation, is Rac1, p22-phox, p47-phox, and p67-phox dependent [18]. However, Rac1 unlike the other proteins also takes part in the activation of Nox1, another pathway of ROS production [38, 39]. Silencing Rac1 would therefore not only affect Nox2 pathway but other ROS generating pathways, hence the significant reduction in ROS seen in shRac1 group.

Rac1, a multifunctional protein, has been studied to play a critical role in cytoskeletal dynamics and cell migration [40]. Coherent with our study, we found that cytokine and focal-adhesion pathways were enriched in BMMSCs-shRac1 group in comparison to BMMSCs-shLuci group (Figure 6G). Rac1 is also reported to be associated with cellular processes such as cell proliferation and survival. For instance, Karabiyik et al. [23] observed that inhibiting Rac1 provided a neuroprotective effect accompanied by downregulation of HIF-1 α, a downstream marker of Rac1 after hypoxia [41]. Similarly, our study showed that Rac1 knockdown facilitated BMMSCs survival, an observation consistent with other previous studies which demonstrated decreased proapoptotic marker JNK activity, and hence decrease in BMMSCs death after Rac1 inhibition. Rac1 is also studied to play a vital role in regulating transcription via activation of nuclear factor-κB and β-catenin transcription factor in the nucleus [42, 43]. Phospho-p53 protein promote repair of damaged DNA, cell cycle arrest, and apoptosis [44]. As per our results there was a decrease in phospho-p53/total p53 expression in BMMSCs-shRac1 group, indicating reduction in cell death in this group. Furthermore, the diversity of Rac1 is dependent on the phosphorylation of the different binding sites [22]. For example, AKT phosphorylation of serine71 on Rac1 enhances guanine diphosphate binding [45]. Previous studies have indicated that [28, 46], ROS signaling results in the regulation of AKT-mediated inhibition of apoptosis. However, Cui et al. [24] found that ROS inhibition of AKT signaling markedly reduced BMMSCs proliferation *in vivo*, but did not increase apoptosis. These studies are coherent with our findings, which showed relationship between ROS and AKT. In addition, our results also showed reversed or decreased expression of pro-apoptotic Bax and anti-apoptotic Bcl-2 which further supported the notion that BMMSCs-shRac1 cell line possess antiapoptotic and pro-survival properties. In contrast, other studies have shown that secretion of Rac1 from mesenchymal stem cells have positive effects on epithelial cell viability [47], and that FGF-dependent Rac1 activation promoted cell proliferation in corneal endothelial cells [48]. This observation was in contrast with our findings and may be due to the different cell lines and cell models used.

The ability of BMMSCs to repair tissue and promote recovery of neurological function depends mainly on its ability to suppress inflammation and promote trophic factor production leading to cell survival, regeneration, neurogenesis, and angiogenesis [8]. Till date trophic factors such as BDNF, GDNF, VEGF, NT3, CSF-1, TGFβ, NGF, the basic fibroblast growth factor (bFGF), the EPOR, the CXC chemokine receptor 4 (CXCR4), and the epidermal growth factor (EGF) have been studied [15, 49–51]. Shen et al. [52] demonstrated that GDNF can be released from BMMSCs and plays major role in BAX/BAD-mediated neural remodeling. From the comparison between BMMSCs-shLuci and BMMSCs-shRac1 cells, we observed significant difference in expressions of these factors (*NGF*, *VEGFA*, *EPOR*, *FGF-1*, *IL-18*, and *CSF-1*), but not in the others (*BDNF*, *GDNF*, *IGF-1*, *FGF-2*, *TGFβ-1*, interleukin 6 (*IL-6)*, and *nuclear factor kappa B*). These findings were consistent other studies which reported upregulation of trophic factors in BMMSCs and promotion of functional recovery.

As a member of the platelet-derived growth factor (PDGF)/VEGF growth factor family, VEGF plays an important role in vascular endothelial cell migration and proliferation, and is indispensable for the development of physiological and pathological angiogenesis following an injury [53]. MMP-2 on the other hand is critical for tumor angiogenesis and metastasis, and inhibiting MMP-2 hinders tumor progression [54]. What both genes have in common is the effect in revascularization. Notably from our results, when *VEGFA* or *MMP-2* gene was suppressed, effect of shRac1 on BMMSCs was dramatically observed. Thus, we are of the view that angiogenesis may have been initiated in Rac1 inhibited BMMSCs survival and thereby improving neurological function in BMMSCs-shRac1 implanted TBI mice.

In conclusion, our study demonstrated that transfecting BMMSC with shRac1 significantly enhanced cell survival both under OGD treatment and at early stage post transplantation in TBI model. At a mechanistic level, *VEGFA* and *MMP-2* were identified to play crucial roles in the shRac1-mediated cell survival. In addition, the transplantation of BMMSCs-shRac1 improved neurological functions in TBI mice, thus may be a promising novel TBI therapy. In consideration of the potential oncogenicity and generation of replication-competent lentiviruses [55], new vector such as adenoviruses could be further considered for engineered cells modification.

## MATERIALS AND METHODS

### Cell cultures

HEK293T cell line was purchased from the American Type Culture Collection (ATCC, USA) while BMMSCs were isolated from Sprague Dawley (SD) rats as previously described method [37]. All cells were cultured in the Dulbecco’s modified Eagle’s medium (DMEM; GE Healthcare Life Science, Pittsburgh, PA, USA) supplemented with 10% fetal bovine serum (FBS; Gibco, Thermo Fisher Scientific, Waltham, MA, USA), 100 U/mL penicillin, and 100 μg/mL streptomycin (Gibco Life Technologies, Darmstadt, Germany), and incubated in a 37° C, 5% CO2 thermostatic incubator. Adherent cells (cell density ~90%) were trypsinized and subcultured. The cells were trypsinized with 0.25% trypsin solution for 3 min at 37° C and re-seeded to a culture dish, or cryopreserved at -80° C in a complete medium containing 10% dimethyl sulfoxide.

### Construction of plasmid, lentivirus production and efficiency determination

Polymerase chain reaction (PCR) was used to generate a lentiviral construct containing miR30 regulatory sequences as described [56, 57] with primers and template sequences as listed in Supplementary Table 1. Restriction enzyme digest and sequencing were performed to confirm all plasmids [56, 57]. Briefly, the lentiviral vectors and the packaging plasmids (pMDL, VSV-G, and pREV) were co-transduced into the HEK293T cells, and the supernatant was collected at the 24 h and 48 h time points. Supernatants containing lentivirus were filtered through a 0.22-μm filter before being used for transfection or stored at -80° C. The filtered lentivirus supernatants were added to the culture dish with complete medium in a 1:1 ratio. The efficiency of infection was measured by detecting mCherry fluorescence (rabbit, Affinity Biosciences, #T0090, 1:200) at 3 days post-infection. For each group, we evaluated three different wells, with five random views of each well being captured and averaged for statistical analysis. Infection efficiency (%) was calculated according to the ratio of mCherry (+)/4′,6-diamidino-2-phenylindole (DAPI (+)).

### OGD

To explore the effects of silencing different genes, we established an OGD model of BMMSCs to observe protein expression, ROS production, apoptosis, and cell growth. Briefly, the BMMSCs were cultured in a glucose-free DMEM (Gibco, Thermo Fisher Scientific, Suzhou, China) at 37° C in an anaerobic incubator (Thermo Fisher Scientific, Marietta, OH, USA) containing 5% CO2 and 1% O2 for 12 h or 18 h. Subsequently, cells were transferred to a normoxic incubator and cultured in a complete medium for another 24 h.

### CCK-8 assays

To assess the effect of gene silencing on BMMSCs viability, CCK-8 assay was utilized to test the relative survival rates of different transfected cell lines. Different BMMSCs cell lines were seeded at 1x10^4^ cells/well into 96-well plates and cultured under OGD condition for 12 h or 24 h. CCK-8 solution (10 μL; Dojindo Molecular Technologies, Inc., Kumamoto, Japan) was subsequently added to each well, and the plates were re-incubated for 3 h at 37° C in a normoxic incubator. To calculate the cell growth rate, absorbance at 450 nm was measured using a microplate reader (Spectramax 190, Molecular Devices, California, USA). Each group had corresponding duplicate wells. The relative cell viability was calculated according to the equation: cell viability (100%) = (experimental group - blank) / (control group - blank) *100%.

### Trypan blue staining

Cells treated with OGD were harvested and re-suspended with phosphate-buffered saline (PBS), and then stained with 2X trypan blue reagent (Beyotime Institute of Biotechnology, Haimen, China) for 3 min. Once stained, cell smear slides were prepared and photographed under an optical microscope. Viable (trypan blue-free) and dead (trypan blue-stained) cells were statistically analyzed from three random eyepiece fields per slide and experiment repeated thrice per each cell line.

### Cell apoptosis analysis by flow cytometry

At the end of OGD treatment, 1x10^6^ cells were washed with cold PBS thrice and centrifuged for 5 min. The pellets were then resuspended in 500 μL binding buffer with 5 μL annexin V and 10 μL propidium iodide for 15 min. Apoptotic cells were detected by flow cytometry within one hour, and the apoptotic rate was analyzed by FlowJo_V10 software.

### Measuring ROS production

Intracellular ROS levels were determined by flow cytometry. The medium was removed, and cells were gently washed twice with PBS. Cells were then incubated with DCFH-DA probe (10 μM; Sigma-Aldrich, Merck KGaA, Germany) for 20 min at 37° C in the dark, and were quantified by flow cytometry within 1 h of DCFH-DA treatment at excitation/emission of 488 nm/525 nm.

### qRT-PCR

qRT-PCR was used to assess the relative expressions of several genes in BMMSCs. Total RNA from BMMSCs was isolated using the TRIzol reagent (Thermo, Waltham, MA, USA), and 1 μg RNA per sample was reverse transcribed using the SuperScript II RT system (Thermo, Waltham, MA, USA). Primers of the target and the housekeeping (GAPDH) genes were designed with the Primer Premier 6.0 software and synthesized by Sangon Biotech (Shanghai, China), as sequences in Supplementary Table 2. qRT-PCR was performed using the SYBR® Fast qPCR Mix (Takara, Dalian, China). The cycle threshold (Ct) value of the target gene was normalized to that of the internal control gene, and the relative change in gene expression was calculated using the formula 2−ΔΔCt.

### Ki67 immunofluorescence staining

Ki67 immunofluorescence staining of cells cultured on coverslips was done to measure BMMSCs proliferation post-OGD. Cells were gently washed twice with PBS, fixed with 4% paraformaldehyde for 15 min at room temperature, and then rewashed thrice with PBS (5 min each). They were then permeabilized with 0.5% Triton X-100 for 20 min, washed thrice with PBS (5 min each), and blocked with 5% bovine serum albumin (BSA; Biofroxx GmbH, Hessen, Germany) for a minimum of 30 min. The blocking solution was discarded, and cells incubated with anti-Ki67 primary antibody (rabbit polyclonal, Proteintech, 27309-1-AP, 1:200) overnight at 4° C, and then with goat anti-rabbit IgG (H+L) DyLight 488 secondary antibody (Bioworld, BS10017, 1:500). The nuclei were stained with DAPI solution (Biosharp, BL105A) and imaged with an inverted fluorescence microscope (Leica Microsystems, Germany). The rate of proliferation was evaluated as the ratio of Ki67-positive (+) cells to the total number of cells (DAPI (+)) per field using the ImageJ software (NIH). For each slide, five random views were captured and averaged for statistical analysis.

### Western blots analysis

Western blotting was performed to analyze the expressions of multiple proteins according to the standard protocols described in our previous research [58]. Briefly, cells were harvested and lysed in the radioimmunoprecipitation assay (RIPA) buffer containing phenylmethylsulfonyl fluoride (PMSF) for 30 min on ice. Total protein concentration was quantified using the Pierce™ bicinchoninic acid assay (BCA) protein assay kit (Thermo Fisher, Waltham, MA, USA). Protein samples were separated on a 10% or 12% SDS-polyacrylamide gel by electrophoresis and then transferred onto 0.22-μm or 0.45-μm Polyvinylidene difluoride (PVDF) membranes. Membranes were blocked with 5% BSA or skim milk for a minimum of 2 h at room temperature, incubated with primary antibody at 4° C overnight, followed by secondary antibody for 1 h at room temperature. Image Lab 3.0 was used for quantitative analysis. For statistical analysis, the mean value from four independent repeats of same experiment was calculated. The following primary antibodies were used at the indicated concentrations: Rac1 (mouse monoclonal, Proteintech, 66122-1-Ig, 1:1000), p22-phox (rabbit polyclonal, Bioss, bs-3879R, 1:1000), p47-phox (rabbit polyclonal, Bioss, bs-6966R, 1:1000), p67-phox (rabbit polyclonal, Proteintech, 15515-1-AP, 1:1000), JNK (rabbit polyclonal, Proteintech, 51151-1-AP, 1:1000), phospho-JNK (rabbit polyclonal, Affinity Biosciences, #AF3318, 1:1000), AKT (rabbit polyclonal, Proteintech, 10176-2-AP, 1:1000), phospho-AKT (rabbit polyclonal, Affinity Biosciences, #AF0016, 1:1000), Bax (rabbit polyclonal, Proteintech, 50599-2-Ig, 1:1000), Bcl-2 (rabbit polyclonal, Proteintech, 12789-1-AP, 1:1000), p53 (rabbit polyclonal, Affinity Biosciences, #AF0879, 1:1000), phospho-p53 (Ser15) (rabbit polyclonal, Affinity Biosciences, #AF3075, 1:1000), VEGFA (rabbit polyclonal, Proteintech, 26157-1-AP, 1:1000), MMP2 (rabbit polyclonal, Proteintech, 10373-2-AP, 1:1000) and beta (β)-actin (rabbit polyclonal, Abcam, ab8227, 1:1000).

### TBI model establishing and BMMSCs transplantation

C57BL/6 male mice (weighing between 15-20 g; specific-pathogen-free (SPF) grade) were purchased from the Shanghai Weitong Lihua Laboratory Animal Technology Co. Ltd. Mice were maintained at the First Affiliated Hospital of the Wenzhou Medical University Animal Center in a 12-h light/12-h dark cycle, with free access to food and water. Mice were anesthetized with 100 mg/kg ketamine hydrochloride (Ketanest, Pfizer, Germany) and 16 mg/kg xylazine hydrochloride (Rompun 2%, Bayer, Germany), and were fixed to the stereotaxic frame (KOPF, Tujunga, CA, USA) in a prone position. TBI was induced according to a previously described protocol [37]. Briefly, using an Impact One™ Stereotaxic Impactor for a controlled cortical impact (Leica, USA) with a 3-mm diameter impact tip, animals received a unilateral blow to the parietal cerebrum with a maximum depression depth of 3 mm, at an impact velocity of 4 m/s and angle of 90°. The impact area was 2.5 mm from the sagittal suture (lateral to the right side) and 3.5 mm posterior to the bregma. Mice were randomly assigned to four groups post-TBI: the TBI group (injected with 5 μL PBS); the TBI+BMMSCs group (BMMSCs transplantation); the TBI+BMMSCs-shLuci group (transplanted with the shLuci-transfected BMMSCs); and the TBI+BMMSCs-shRac1 group (transplanted with the shRac1-transfected BMMSCs). BMMSCs cell suspensions were prepared by harvesting adherent cells with 0.25% trypsin solution, which were then resuspended with cold PBS and placed on ice for subsequent transplantation. At 24 h post-TBI, 5 μL cells (5 x 10^5^ cells) in PBS was slowly injected (1 μL/min) using a microinjection needle (Hamilton7000, Switzerland) into the center of the lesion. It was necessary to keep needle in place for 3 min before removing the needle. To reduce bias, we performed stem cell transplantation on only three mice at a time, and the total transplantation time was controlled within half an hour. In addition, due to the proximity of the animal center to the cell culture chamber, and division of labor, stem cell transplantation could be completed within half an hour after collection. All animal experimental procedures were carried out in accordance with the guidelines of the Animal Care and Use Committee of the Wenzhou Medical University (China).

### Assessment of lesion volume

To further evaluate the size of the brain lesions, hematoxylin and eosin (H&E)-stained of the coronal tissue sections were prepared as per a previously published method [37]. Mice at day 7 or day 21 post-transplantation were sacrificed, and infused with saline and cold 4% paraformaldehyde through the left ventricle. The brains were extracted and fixed in 4% paraformaldehyde at 4° C for 24 h, dehydrated in 50%-100% ethyl alcohol, made transparent with xylene before being embedded in paraffin. From the center of the lesion, 5 consecutive coronal sections were sliced anteriorly and posteriorly at 1mm interval for staining and analysis. The sections were stained with a hematoxylin and eosin staining kit (Solarbio Science and Technology Co., Beijing, China) according to the kit instructions, and imaged with an inverted fluorescence microscope (Leica Microsystems, Germany). We assessed the lesion volume ratio using the calculation: a section of the affected ipsilateral side divided by the section from the healthy contralateral side.

### Determination of brain water content

After BMMSCs transplantation 7 days or 21 days, mice were sacrificed and decapitated. The wet mass of initial brain was weighed and later reweighed for the dry mass after 2 h of baking. Brain water content is calculated based on following algorithms: Brain water content (%) = (wet mass - dry mass)/wet mass * 100%.

### Behavioral evaluations

Double-blind studies using the mNSS and the rotarod test were conducted at day -1 (day before TBI), day 0, day 1, day 3, day 7, day 14, and day 21 post-TBI. For mNSS, motor (muscle strength and movement), sensory (touch, visual and proprioceptive), balance, and reflex tests were conducted based on a previously published method [59], with a score of 0 indicating normal and 18 indicating a serious neurologic deficit. The rotarod test was used to assess the motor function [60], which recorded the time that a mouse remained on a rod that rotated at an increasing velocity (4 r/min to 40 r/min). For either the mNSS assessment or the rotarod test, the mice were pre-trained before TBI. For behavioral statistics, we evaluated eight mice in each group, and also eight mice at each time point.

### Survival and distribution of BMMSCs and neuron

The survival and distribution of the transplanted BMMSCs and neurons were investigated using immunofluorescence staining. Mice were sacrificed 3 days, 7 days, and 21 days after transplantation, and infused with saline and cold 4% paraformaldehyde. The brains were further fixed in 4% paraformaldehyde at 4° C for 24 h and then stored in a 30% sucrose solution for 48 h-72 h at 4° C. Preprocessed brain tissue was embedded in an optimal cutting temperature (OCT) reagent (Sakura Finetec, Tokyo, Japan), and frozen sections were prepared using a microtome (Leica, Germany). Around the central area of TBI, six slides from each brain were selected for staining. These sections were incubated with primary anti-mCherry (rabbit, Affinity Biosciences, #T0090, 1:200) and anti-NeuN (rabbit, Abcam, ab177487, 1:200) respectively, and corresponding secondary antibody, followed by counterstain with DAPI, and imaged using a fluorescence microscope. For statistical analysis, five unduplicated random fields from each slide were imaged for counting. ImageJ (NIH) was used to quantify the average number of positive cells per square millimeter. The survival of BMMSCs and neurons were evaluated by the amount of mCherry (+) cells and ratio of NeuN (+)/DAPI (+) respectively.

### Bioinformatics analyses

Total RNA was isolated from different transfected BMMSCs lines (parent BMMSCs, BMMSCs-shLuci, BMMSCs-shRac1, BMMSCs-shLuci+OGD 12 h, and BMMSCs-shRac1+OGD 12 h). Sequencing and data analysis for RNA-seq were performed at ShuPu (Shanghai) BIOTECHNOLOGY LLC. Differences in the mRNA expressions were displayed on heatmaps. The GO and KEGG analyses were performed to investigate the biological pathways associated with the enrichment of up- and down-regulated genes in different BMMSCs datasets. The top 10 GO and KEGG terms were ranked based on the enrichment scores (-log10 p-value) and fragments per kilobase million (FPKM) ≥ 0.5 cut-off values. A protein-protein interaction network of differentially expressed genes (DEGs) was constructed with the STRING online program (http://string-db.org/). Among the DEGs, 10 hub genes were identified by the Cytoscape V3.7.2 software (the Cytoscape Consortium, San Diego, CA, USA).

### Statistical analysis

All data have been expressed as the mean ± standard deviation (SD) and analyzed with the GraphPad Prism 5.0 software (GraphPad, USA). The Student’s t-test was used to compare two experimental groups. Differences between three or more groups were assessed by one-way or two-way ANOVA, followed by the Bonferroni correction. In our study, a value of p ≤ 0.05 was considered significant.

### Availability of data and materials

All data generated or analyzed during this study are included in the published article.

### Ethics approval and consent to participate

All animal experiments performed were in accordance with the institutional guidelines for animal research and approved by the Animal Care Committee of Wenzhou Medical University (China).

## Supplementary Material

Supplementary Figures

Supplementary Tables 1 and 2

Supplementary Table 3
