# Supplementary material for: Transplanting Rac1-silenced bone marrow mesenchymal stem cells promote neurological function recovery in TBI mice
Source: Aging (Albany NY). 2020 Dec 19;13(2):2822–50. doi: 10.18632/aging.202334 (PMC7880331; doi:10.18632/aging.202334)
Supplement: Supplementary Figures [file aging-13-202334-s001.pdf]

SUPPLEMENTARY FIGURES

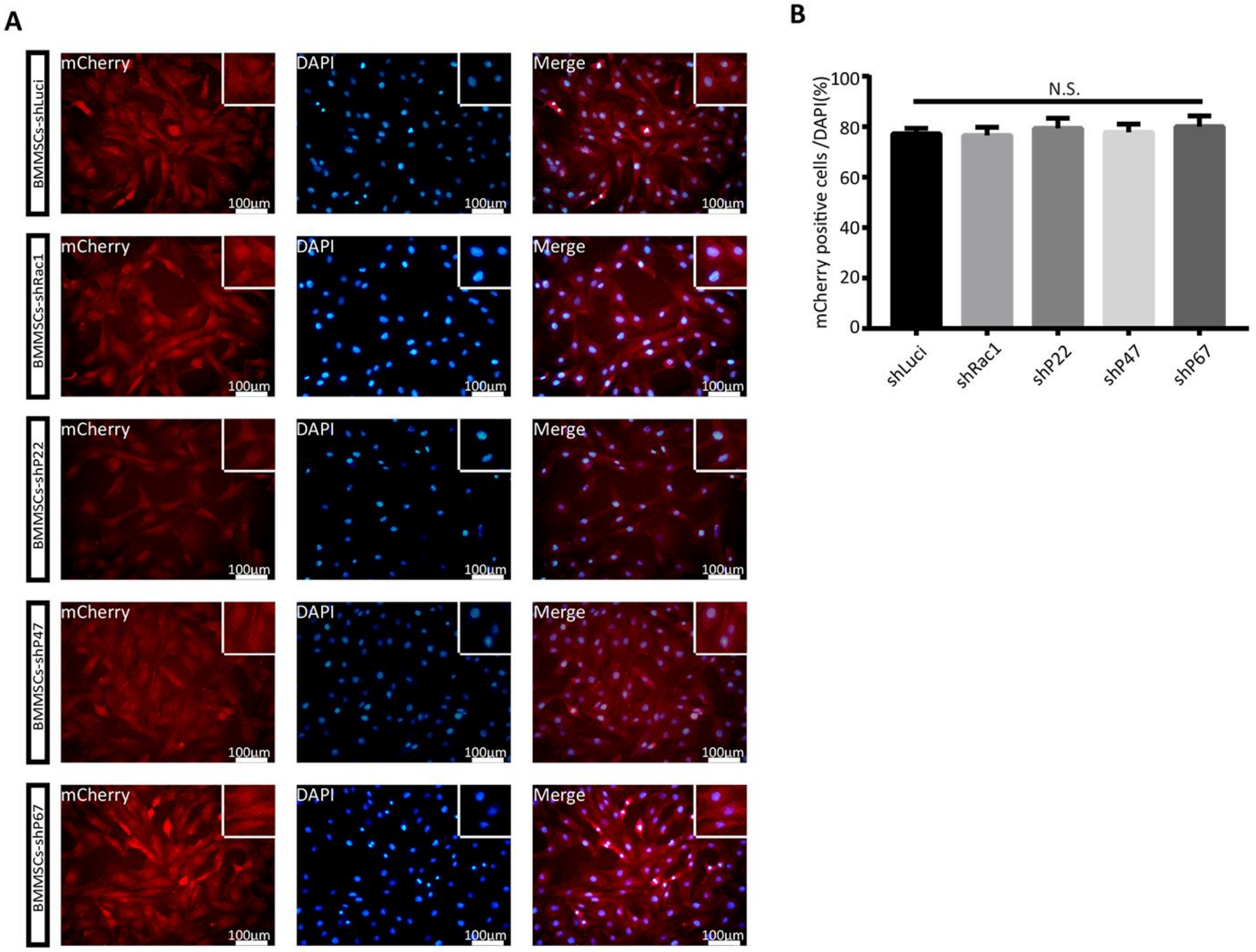

Supplementary Figure 1. shLuci, shRac1, shP22, shP47, or shP67 lentivirus effectively infectes BMMSCs. (**A**) Display images of BMMSCs transfected with lentivirus (shLuci, shRac1, shP22, shP47, or shP67) 3 days post-transfection, mCherry (red) indicates positively transfected cells. The upper right corner insert shows a zoomed-in image of the local area with scale bar of 100 µm. (**B**) The mCherry (+)/DAPI ratio of shLuci-, shRac1-, shP22-, shP47-, and shP67-transfected cells showed that the transfection efficiency was close to 80% (n = 3). Data are presented as mean ± SD.

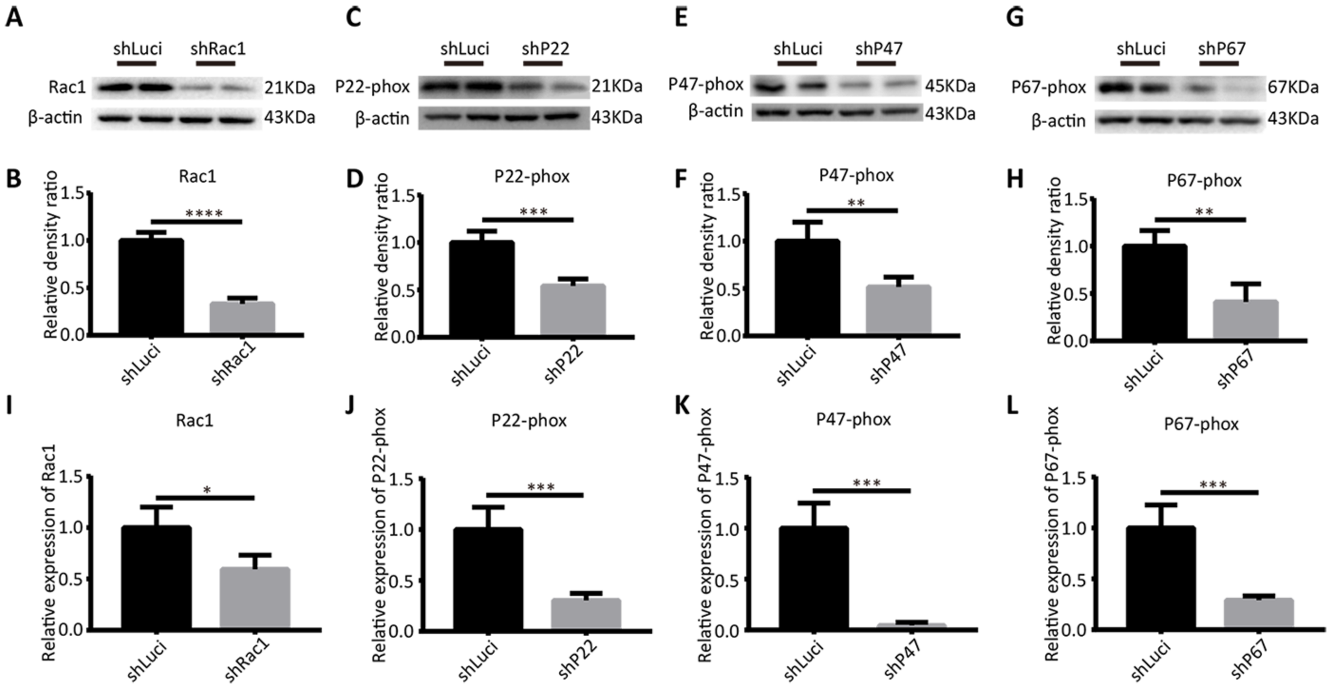

Supplementary Figure 2. shRac1, shP22, shP47, and shP67 significantly knock down the gene expressions. (**A**–**H**) Western blot analyses of Rac1, p22-phox, p47-phox and p67-phox expressions in BMMSCs transfected with shRac1, shP22, shP47, shP67, and shLuci lentivirus and after OGD 12 h (**P < 0.01, ***P < 0.001, ****P < 0.0001, statistically analyzed by the Student's t-test, n = 4). (**I**–**L**) qRT-PCR analysis of *Rac1*, *p22-phox*, *p47-phox*, and *p67-phox* mRNA expressions in BMMSCs transfected with shRac1, shP22, shP47, shP67, and shLuci lentivirus post-OGD 12 h. (*P < 0.05, ***P < 0.001, by the Student's t-test, n = 3). Data are presented as mean ± SD.

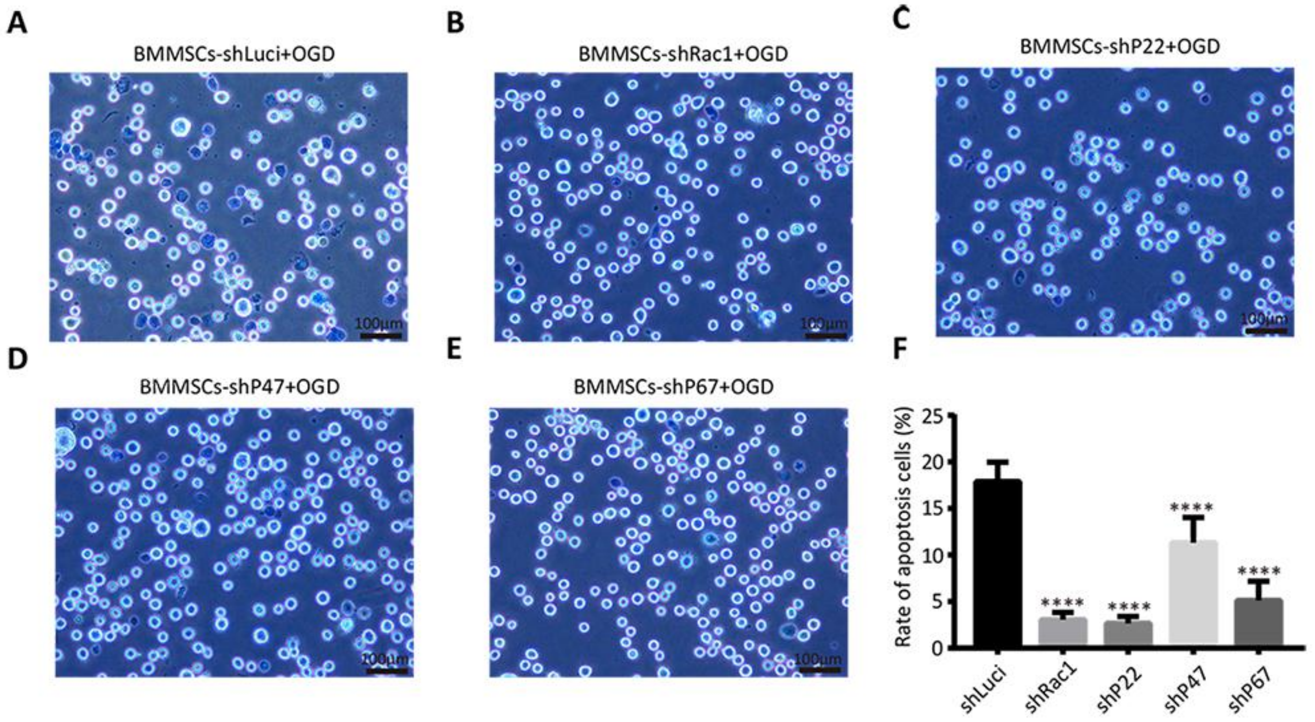

Supplementary Figure 3. shRac1, shP22, shP47, and shP67 promote cell survival of BMMSCs after an OGD treatment. (**A**–**E**) Images representing trypan blue-stained BMMSCs transfected with shLuci, shRac1, shP22, shP47, and shP67 lentiviruses and after OGD 12 h treatment, with scale bar of 100 μm. (**F**) Quantification and statistical analysis of A-E. (****P < 0.0001, statistically analyzed by one-way ANOVA followed by the Bonferroni correction, n = 3). Data are presented as mean ± SD.

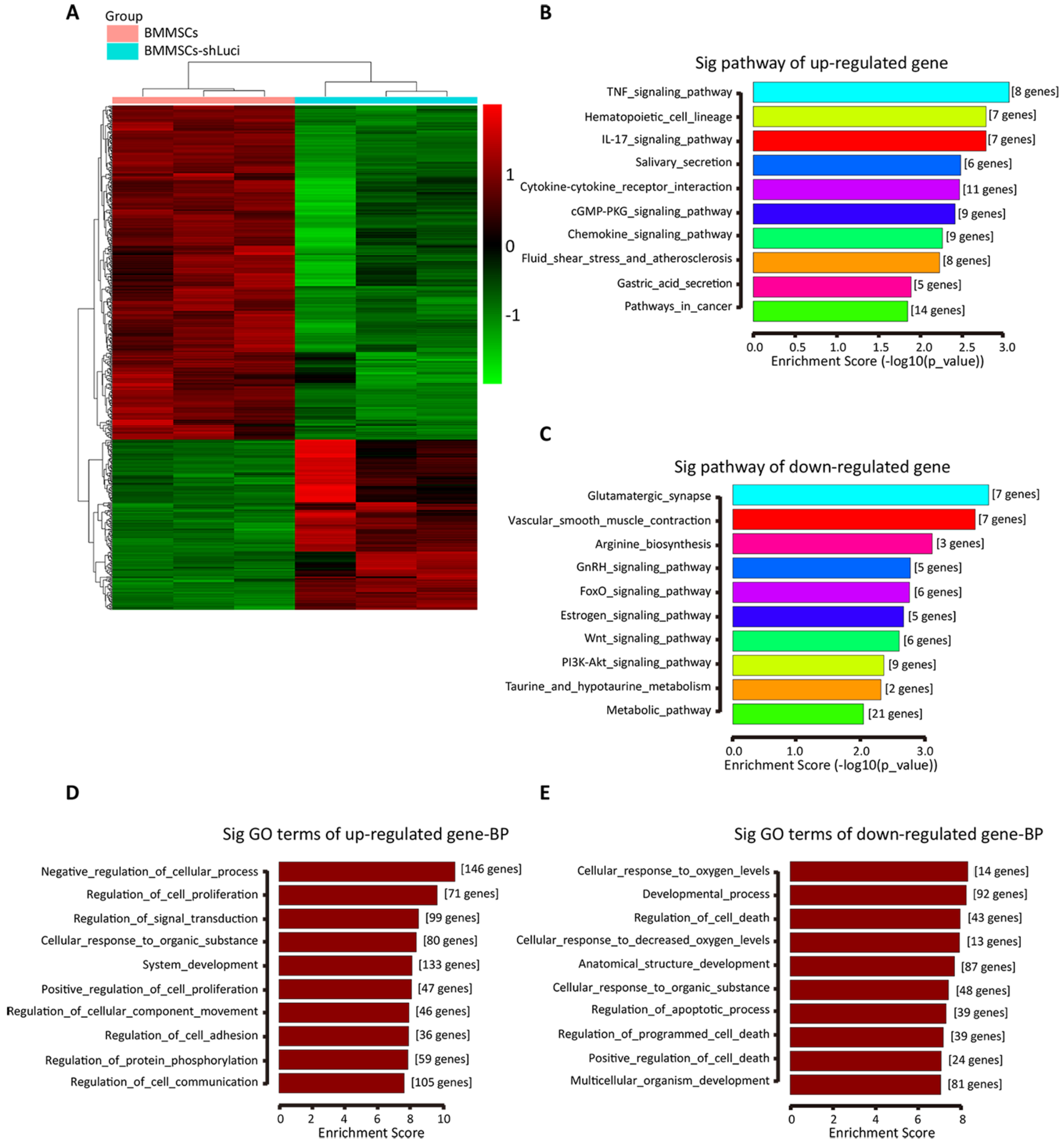

Supplementary Figure 4. Differences in gene expression between parental BMMSCs and BMMSCs-shLuci cells. (**A**) Heatmap showing differences in gene expression between parental BMMSCs and BMMSCs-shLuci cells based on RNA-Seq data. Red color indicates high expression, and green color indicates low expression. (**B**, **C**) The KEGG enrichment pathway analysis of differential gene expression in parental BMMSCs and BMMSCs-shLuci cells based on the RNA-Seq data. (**D**, **E**) Analysis of differential expression of genes associated with different biological processes in parental BMMSCs and BMMSCs-shLuci cells based on the RNA-Seq data. Data are presented as mean ± SD.

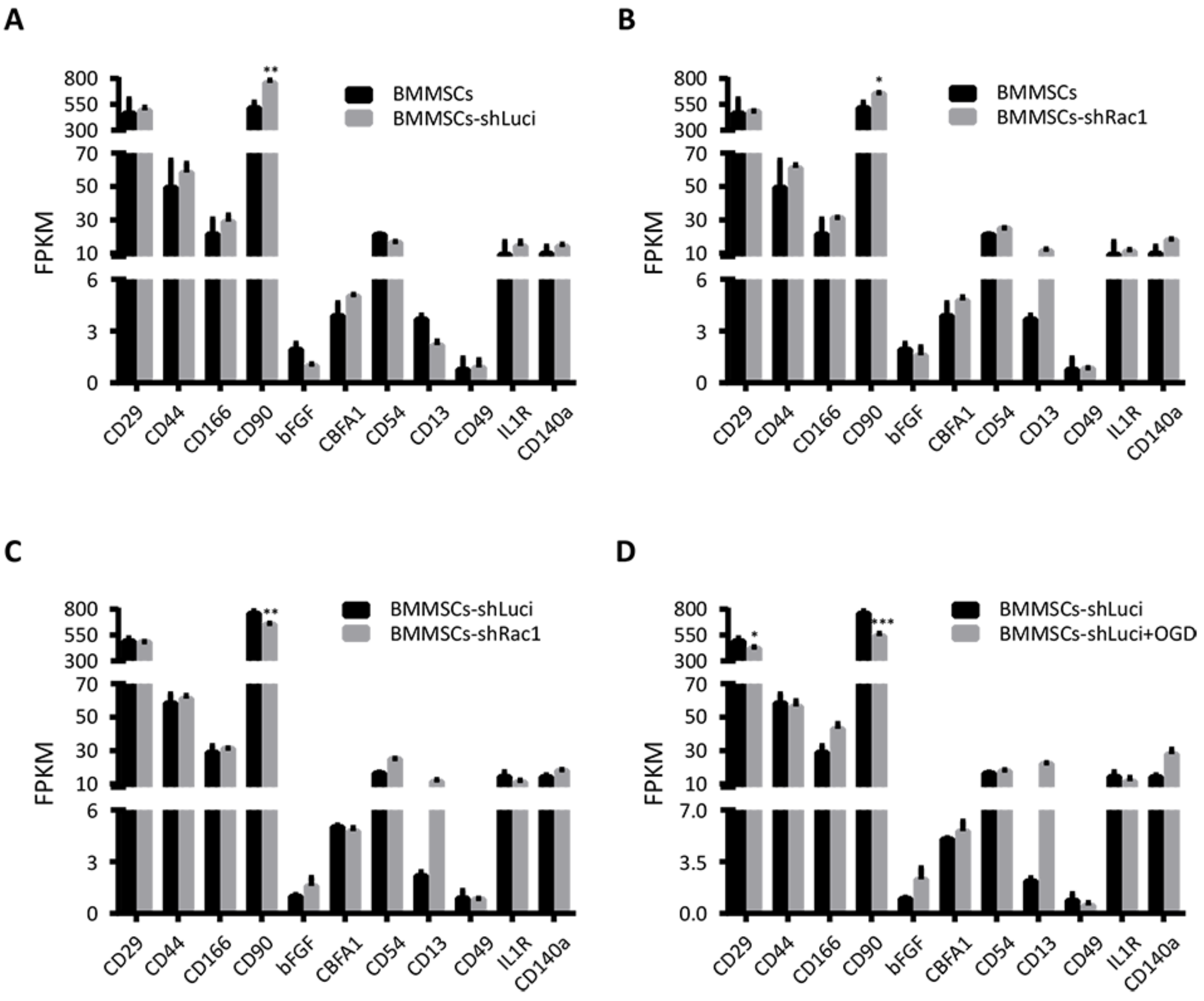

Supplementary Figure 5. The differential expression of typical BMMSCs markers in different BMMSCs RNA-seq datasets. The differential expression of 11 typical BMMSCs markers in: (**A**) BMMSCs versus BMMSCs-shLuci; (**B**) BMMSCs versus BMMSCs-shRac1; (**C**) BMMSCs-shLuci versus BMMSCs-shRac1; (**D**) BMMSCs-shLuci versus BMMSCs-shLuci+OGD (*P < 0.05, **P < 0.01, ***P < 0.001, statistically analyzed by the Student's t-test, n = 3). Data are presented as mean ± SD.
