# Supplementary material for: Transplanting Rac1-silenced bone marrow mesenchymal stem cells promote neurological function recovery in TBI mice
Source: Aging (Albany NY). 2020 Dec 19;13(2):2822–50. doi: 10.18632/aging.202334 (PMC7880331; doi:10.18632/aging.202334)
Supplement: Supplementary Tables 1 and 2 [file aging-13-202334-s002.pdf]

SUPPLEMENTARY TABLES

Supplementary Table 1. Primer sequence of inserted fragment aiming at target genes.

Identifier	Sequence
shRNA forward primer	5'-TGAACTCGAGAAGGTATATTGCTGTTGACAGTGAGCG-3'
shRNA reverse primer	5'-TCTCGAATTCTAGCCCCTTGAAGTCCGAGGCAGTAGGC-3'
Template Rac1 shRNA 1	5'-TGCTGTTGACAGTGAGCGACAAGTTTTTGTTACAGATTAATAGTGAAGCCAC AGATGTATTAATCTGTAACAAAAACTTGGTGCCTACTGCCTCGGA-3'
Template Rac1 shRNA 2	5'-TGCTGTTGACAGTGAGCGCCAGACAGTATTTTGACTAATATAGTGAAGCCAC AGATGTATATTAGTCAAAATACTGTCTGTTGCCTACTGCCTCGGA-3'
Template P22 shRNA 1	5'-TGCTGTTGACAGTGAGCGTGGAGCGGTGTGGACAGAAGTATAGTGAAGCCA CAGATGTATACTTCTGTCCACATCGCTCCATGCCTACTGCCTCGGA-3'
Template P22 shRNA 2	5'-TGCTGTTGACAGTGAGCGTGGTGATGCCCACTGTAATAAATAGTGAAGCCAC AGATGTATTTATTACAGTGAGCAACACCATGCCTACTGCCTCGGA-3'
Template P47 shRNA 1	5'-TGCTGTTGACAGTGAGCGACGAGATCTACGAGTTCCATAATAGTGAAGCCACA GATGTATTATGGAACTCGTAGATCTCGGTGCCTACTGCCTCGGA-3'
Template P47 shRNA 2	5'-TGCTGTTGACAGTGAGCGCACGCAGGTGAACCGTATGTAATAGTGAAGCCACA GATGTATTACATACGGTTCACCTGCGTATGCCTACTGCCTCGGA-3'
Template P67 shRNA 1	5'-TGCTGTTGACAGTGAGCGCTCGGAACATGGTGTCTAAGAATAGTGAAGCCACA GATGTATTCTTAGACACCATGTTCCGAATGCCTACTGCCTCGGA-3'
Template P67 shRNA 2	5'-TGCTGTTGACAGTGAGCGTCAGGAAGATACCTCCCTGGAATAGTGAAGCCACA GATGTATTCTGGAGAGGTATCTTCCTGGTGCCTACTGCCTCGGA-3'
Template Vegfa shRNA 1	5'-TGCTGTTGACAGTGAGCGCTAGAGAATTCTACATACTAAATAGTGAAGCCACA GATGTATTTAGTATGTAGAATTCTCTATTGCCTACTGCCTCGGA-3'
Template Vegfa shRNA 2	5'-TGCTGTTGACAGTGAGCGACAACTTGTGTTTGTATATAAATAGTGAAGCCACA GATGTATTTATATACAAACACAAGTTGCTGCCTACTGCCTCGGA-3'
Template Ctgf shRNA 1	5'-TGCTGTTGACAGTGAGCGCACAGTTATCTAAGTTAATTTATAGTGAAGCCACA GATGTATAAATTAACTTAGATAACTGTATGCCTACTGCCTCGGA-3'
Template Ctgf shRNA 2	5'-TGCTGTTGACAGTGAGCGAACCTTTCTAGTTGAAAATAAATAGTGAAGCCACA GATGTATTTATTTTCAACTAGAAAGGTGTGCCTACTGCCTCGGA-3'
Template Mmp2 shRNA 1	5'-TGCTGTTGACAGTGAGCGATGGTGAGATCTTCTTCTTCAATAGTGAAGCCACA GATGTATTGAAGAAGAAGATCTCACCACTGCCTACTGCCTCGGA-3'
Template Mmp2 shRNA 2	5'-TGCTGTTGACAGTGAGCGAACCTGGTGACTTCAGATTTAATAGTGAAGCCACA GATGTATTAAATCTGAAGTCACCAGGTGTGCCTACTGCCTCGGA-3'
Template Ccl2 shRNA 1	5'-TGCTGTTGACAGTGAGCGCTCCAAGGTATTGTTTAAAATATAGTGAAGCCACA GATGTATATTTTAAACAATACCTTGGAATGCCTACTGCCTCGGA-3'
Template Ccl2 shRNA 2	5'-TGCTGTTGACAGTGAGCGCACCACCTCAAGCACTTCTGTATAGTGAAGCCACA GATGTATACAGAAGTGCTTGAGGTGGTTTGCCTACTGCCTCGGA-3'
Template Tgfb2 shRNA 1	5'-TGCTGTTGACAGTGAGCGAAAGCTTGTGTTCTGTTTGTTATAGTGAAGCCACAG ATGTATAACAAACAGAACACAAGCTTGTGCCTACTGCCTCGGA-3'
Template Tgfb2 shRNA 2	5'-TGCTGTTGACAGTGAGCGCAGCTTGTGTTCTGTTTGTTAATAGTGAAGCCACAG ATGTATTAACAAACAGAACACAAGCTTTGCCTACTGCCTCGGA-3'
Template Tgfb3 shRNA 1	5'-TGCTGTTGACAGTGAGCGCCAGAATCTTTGTATAAATAAATAGTGAAGCCACA GATGTATTTATTTATACAAAGATTCTGATGCCTACTGCCTCGGA-3'
Template Tgfb3 shRNA 2	5'-TGCTGTTGACAGTGAGCGACACGGTGCTTGGACTATACAATAGTGAAGCCACA GATGTATTGTATAGTCCAAGCACCGTGCTGCCTACTGCCTCGGA-3'
Template Edn1 shRNA 1	5'-TGCTGTTGACAGTGAGCGCACCGAGCACATTGACTACAGATAGTGAAGCCACA

	GATGTATCTGTAGTCAATGTGCTCGGTTTGCCTACTGCCTCGGA-3'
Template Edn1 shRNA 2	5'-TGCTGTTGACAGTGAGCGGCCCAAAGTACCATGCAGAAAGTAGTGAAGCCAC AGATGTATTTTCTGCATGGTACTTTGGGCTGCCTACTGCCTCGGA-3'
Template GAS6 shRNA 1	5'-TGCTGTTGACAGTGAGCGACCGTGATTAGACTACGCTTCATAGTGAAGCCACA GATGTATGAAGCGTAGTCTAATCACGGGTGCCTACTGCCTCGGA-3'
Template GAS6 shRNA 2	5'-TGCTGTTGACAGTGAGCGACCACTCCACAAAGAAGCTCAATAGTGAAGCCAC AGATGTATTGAGCTTCTTTGTAGAGTGGTTGCCTACTGCCTCGGA-3'
Template CXCL1 shRNA 1	5'-TGCTGTTGACAGTGAGCGCTCAAAGGACTGTTACAAATGATAGTGAAGCCACA GATGTATCATTTGTAACAGTCCTTTGAATGCCTACTGCCTCGGA-3'
Template CXCL1 shRNA 2	5'-TGCTGTTGACAGTGAGCGCAGGACTGTTACAAATGAAATATAGTGAAGCCACA GATGTATACTTCATTTGTAACAGTCCTTTGCCTACTGCCTCGGA-3'
Template Thbs1 shRNA 1	5'-TGCTGTTGACAGTGAGCGAACCCATCTATGACAAAACCTATAGTGAAGCCACA GATGTATAGGTTTTGTCATAGATGGGTCTGCCTACTGCCTCGGA-3'
Template Thbs1 shRNA 2	5'-TGCTGTTGACAGTGAGCGCTACGAGTGCCGAGACTCCTAATAGTGAAGCCACA GATGTATTAGGAATCTCGACACTCGTATTGCCTACTGCCTCGGA-3'

Note: Sense shRNA and antisense shRNA sequences are underlined.

Supplementary Table 2. Primer sequence of target genes for qPCR experiments.

Gene symbol	Forward primer sequence	Reverse primer sequence
Rac1	5'-ACATCCCCACCGTCTTTGAC-3'	5'-ACCACATGTGTCTCCAACTGT-3'
P22	5'-CCTCCACTTACTGCTGTCCG-3'	5'-AATGGGAGTCCACTGCTCAC-3'
P47	5'-AGAAACGCTTCGTCCCCAG-3'	5'-TCCTTTAACATTTTATGGAACTCGT-3'
P67	5'-CTGAGCAGGCCTTCACCAAA-3'	5'-GCCTTCCTACCCACAGAAGC-3'
Vegfa	5'-TGGAGCGTATGTGACAAGCC-3'	5'-TTCTGTCGACGGTGACGATG-3'
Ctgf	5'-CCCTAGCTGCCTACCGACT-3'	5'-TTGGTAACCCGGGTGGAGAT-3'
Mmp2	5'-TCCCCACGAAGCCTTGTTTA-3'	5'-TCATAATCCTCGGTGGTGCC-3'
Ccl2	5'-AGAAACCAGCCAACTCTCACT-3'	5'-GCATTAACTGCATCTGGCTGAG-3'
Tgfb2	5'-CTCTGTGGGTACCTTGATGCC-3'	5'-GCAAGCGAAAGACCCTGAAC-3'
Tgfb3	5'-ACACAGCGCAGTGAGTTCAT-3'	5'-TGTGTGAACCCAGGAACGAG-3'
Edn1	5'-AGCCCTTCTAGGTCTAAGCGA-3'	5'-CATGGTACTTTGGGCTCGGA-3'
GAS6	5'-GGATGGTACCAAGGGCCAGA-3'	5'-AGTCACTTGTACATCTGGCAGG-3'
CXCL1	5'-CCGAAGTCATAGCCACACTCA-3'	5'-ACGCCATCGGTGCAATCTAT-3'
Thbs1	5'-CGAGTCGCAAAGGGAGATGT-3'	5'-ACGTTGGTTGAACTGGAGCA-3'
GAPDH	5'-GACATGCCGCCTGGAGAAAC-3'	5'-AGCCCAGGATGCCCTTTAGT-3'
