# Supplementary material for: Transplanting Rac1-silenced bone marrow mesenchymal stem cells promote neurological function recovery in TBI mice
Source: Aging (Albany NY). 2020 Dec 19;13(2):2822–50. doi: 10.18632/aging.202334 (PMC7880331; doi:10.18632/aging.202334)
Supplement: Supplementary Table 3 [file aging-13-202334-s003.docx]

**Supplementary Table 3.** **317 detected up-regulate genes between OGD+BMSC-shLuci and OGD+BMSC-shRac1 group.**

| **Track_id** | **Gene_Name** | **Fold_Change** | **Track_id** | **Gene_Name** | **Fold_Change** |
| --- | --- | --- | --- | --- | --- |
| ENSRNOG00000005695 | Mgp | 6.870700836 | ENSRNOG00000010146 | Ndn | 1.733043143 |
| ENSRNOG00000032300 | Rpl39l | 6.654305712 | ENSRNOG00000000500 | Scube3 | 1.730889197 |
| ENSRNOG00000014361 | Edn1 | 5.651884333 | ENSRNOG00000013867 | Fgf1 | 1.727238572 |
| ENSRNOG00000004708 | Aard | 4.017990626 | ENSRNOG00000032048 | Zfp462 | 1.72367141 |
| ENSRNOG00000007002 | Lif | 3.840618273 | ENSRNOG00000013707 | Spata13 | 1.723115844 |
| ENSRNOG00000030034 | Sox11 | 3.734763305 | ENSRNOG00000010047 | Ddit4l | 1.722575735 |
| ENSRNOG00000005727 | Galnt3 | 3.480074419 | ENSRNOG00000012508 | Slc39a8 | 1.716934074 |
| ENSRNOG00000028624 | Kif26b | 3.282258331 | ENSRNOG00000011526 | Pcsk6 | 1.715778432 |
| ENSRNOG00000010325 | Ptger3 | 3.236672899 | ENSRNOG00000060773 | Sertad4 | 1.714407121 |
| ENSRNOG00000016420 | Serpinb6b | 3.168313522 | ENSRNOG00000043426 | Kyat3 | 1.713521215 |
| ENSRNOG00000030920 | Rtn4r | 3.112364224 | ENSRNOG00000020279 | Syt11 | 1.713005067 |
| ENSRNOG00000015904 | Wfdc1 | 2.994136465 | ENSRNOG00000046202 | Metrnl | 1.711544739 |
| ENSRNOG00000003537 | Spta1 | 2.862914185 | ENSRNOG00000012952 | Lrig1 | 1.70917566 |
| ENSRNOG00000013526 | Rassf4 | 2.859316902 | ENSRNOG00000000551 | RGD1305587 | 1.708444667 |
| ENSRNOG00000006877 | Efnb1 | 2.856975033 | ENSRNOG00000020467 | Nrep | 1.70809391 |
| ENSRNOG00000027024 | Rgs16 | 2.850954364 | ENSRNOG00000027736 | Cnn1 | 1.699361444 |
| ENSRNOG00000014683 | Il1rl2 | 2.799853504 | ENSRNOG00000014008 | Mfsd2a | 1.698748085 |
| ENSRNOG00000009311 | Fstl3 | 2.797127361 | ENSRNOG00000012802 | Tenm3 | 1.69850157 |
| ENSRNOG00000018570 | C1qtnf3 | 2.776684614 | ENSRNOG00000015658 | Sorbs1 | 1.697813358 |
| ENSRNOG00000017209 | Tubb3 | 2.74235402 | ENSRNOG00000013742 | Large1 | 1.695680589 |
| ENSRNOG00000058470 | Col12a1 | 2.737669636 | ENSRNOG00000003069 | Cd38 | 1.695602645 |
| ENSRNOG00000012053 | S100a16 | 2.703593051 | ENSRNOG00000033528 | Tll1 | 1.695415952 |
| ENSRNOG00000024243 | Cadm4 | 2.688108429 | ENSRNOG00000008034 | Tmeff1 | 1.694245949 |
| ENSRNOG00000057880 | Myh11 | 2.634760704 | ENSRNOG00000001158 | Abcg1 | 1.693201914 |
| ENSRNOG00000004281 | Cobl | 2.61514981 | ENSRNOG00000008000 | Syt13 | 1.691608612 |
| ENSRNOG00000009700 | Dync1i1 | 2.607632901 | ENSRNOG00000015036 | Ctgf | 1.691057097 |
| ENSRNOG00000024536 | Ccbe1 | 2.603460329 | ENSRNOG00000054186 | Krtap1-5 | 1.689165907 |
| ENSRNOG00000020851 | Aplp1 | 2.546786377 | ENSRNOG00000007091 | Ly6e | 1.684993863 |
| ENSRNOG00000004303 | Timp3 | 2.532766994 | ENSRNOG00000000142 | Plxdc2 | 1.684513996 |
| ENSRNOG00000002418 | Tgfb2 | 2.510389368 | ENSRNOG00000048302 | LOC100910979 | 1.683473343 |
| ENSRNOG00000010524 | Cryab | 2.504184789 | ENSRNOG00000052758 | Fam49a | 1.682094592 |
| ENSRNOG00000029598 | Robo2 | 2.495083208 | ENSRNOG00000021447 | Prr7 | 1.680158694 |
| ENSRNOG00000010183 | Fam198b | 2.463509813 | ENSRNOG00000010799 | Noct | 1.678434275 |
| ENSRNOG00000019184 | Npr3 | 2.456014927 | ENSRNOG00000017918 | Iglon5 | 1.674817848 |
| ENSRNOG00000025160 | Tmem56 | 2.449650674 | ENSRNOG00000031041 | Rps4y2 | 1.674014288 |
| ENSRNOG00000018366 | RGD1310819 | 2.425104046 | ENSRNOG00000013090 | Gadd45g | 1.671009444 |
| ENSRNOG00000048935 | Tmem45al | 2.418373925 | ENSRNOG00000014367 | Ephb6 | 1.66890399 |
| ENSRNOG00000006052 | Sulf2 | 2.402575976 | ENSRNOG00000021256 | Adra1d | 1.668295879 |
| ENSRNOG00000014574 | Entpd1 | 2.39590168 | ENSRNOG00000001653 | St3gal6 | 1.665860602 |
| ENSRNOG00000030210 | Fndc1 | 2.335825569 | ENSRNOG00000020695 | Tead2 | 1.665277801 |
| ENSRNOG00000058609 | Palmd | 2.261618065 | ENSRNOG00000002775 | Npl | 1.662511188 |
| ENSRNOG00000029401 | Actg2 | 2.258877416 | ENSRNOG00000039902 | Lbh | 1.660037934 |
| ENSRNOG00000009867 | Tgfb3 | 2.256403721 | ENSRNOG00000010302 | Rasl10b | 1.655973018 |
| ENSRNOG00000011927 | Sdc3 | 2.251402511 | ENSRNOG00000049943 | Fam60a | 1.65495963 |
| ENSRNOG00000058645 | Tnc | 2.242467624 | ENSRNOG00000059764 | Snrpn | 1.650205038 |
| ENSRNOG00000014686 | Kcnd3 | 2.23918044 | ENSRNOG00000012260 | Ddx25 | 1.64945453 |
| ENSRNOG00000010392 | Nrg1 | 2.236334596 | ENSRNOG00000014961 | Pdpn | 1.648260078 |
| ENSRNOG00000009773 | Elovl4 | 2.235256807 | ENSRNOG00000014259 | Mycl | 1.645932433 |
| ENSRNOG00000016451 | Cd1d1 | 2.23100049 | ENSRNOG00000054420 | Vdr | 1.644164618 |
| ENSRNOG00000032703 | Rasgrp3 | 2.221756857 | ENSRNOG00000013954 | Alpl | 1.643806038 |
| ENSRNOG00000027096 | Ctsw | 2.193241146 | ENSRNOG00000016538 | Itga8 | 1.640899232 |
| ENSRNOG00000042975 | Tmem45a | 2.178107179 | ENSRNOG00000016243 | Casq2 | 1.639734197 |
| ENSRNOG00000030486 | Prdm6 | 2.176823097 | ENSRNOG00000017253 | Ccdc107 | 1.639076522 |
| ENSRNOG00000011923 | Mgarp | 2.172120523 | ENSRNOG00000014490 | Bdh2 | 1.634110211 |
| ENSRNOG00000023465 | LOC500300 | 2.157576642 | ENSRNOG00000009581 | Lce1m | 1.632021361 |
| ENSRNOG00000048449 | Itgb3 | 2.145058966 | ENSRNOG00000004585 | Tmtc2 | 1.625225857 |
| ENSRNOG00000023148 | Col11a1 | 2.142386648 | ENSRNOG00000059947 | Sdc1 | 1.625172198 |
| ENSRNOG00000006738 | Fbxo32 | 2.136791522 | ENSRNOG00000045829 | Thbs1 | 1.622759025 |
| ENSRNOG00000018770 | Pmaip1 | 2.13241376 | ENSRNOG00000004861 | Itga4 | 1.622160683 |
| ENSRNOG00000012424 | Adam23 | 2.128965359 | ENSRNOG00000014066 | Jade1 | 1.621616652 |
| ENSRNOG00000048056 | Spag4 | 2.11983646 | ENSRNOG00000012521 | Shisa2 | 1.620656919 |
| ENSRNOG00000043219 | Fbn2 | 2.112218329 | ENSRNOG00000038483 | Tnfrsf10b | 1.620520521 |
| ENSRNOG00000007118 | Eva1a | 2.108644197 | ENSRNOG00000005573 | Ntn4 | 1.620500717 |
| ENSRNOG00000015380 | Jup | 2.105734647 | ENSRNOG00000000341 | Nid2 | 1.620080175 |
| ENSRNOG00000018598 | Ankrd1 | 2.090990975 | ENSRNOG00000060687 | Slc24a3 | 1.618637177 |
| ENSRNOG00000017307 | Prss23 | 2.088512916 | ENSRNOG00000017628 | Tagln | 1.617761853 |
| ENSRNOG00000014613 | Ddah1 | 2.079391731 | ENSRNOG00000016571 | Ngf | 1.615756353 |
| ENSRNOG00000007779 | Kank4 | 2.075777065 | ENSRNOG00000019622 | Ackr3 | 1.612507928 |
| ENSRNOG00000061050 | Mboat2 | 2.069322679 | ENSRNOG00000003120 | Prelp | 1.611881501 |
| ENSRNOG00000050792 | Tnfaip6 | 2.060917676 | ENSRNOG00000013589 | Cxcl12 | 1.609287573 |
| ENSRNOG00000000201 | Gsta5 | 2.060190213 | ENSRNOG00000005275 | Shmt1 | 1.608456761 |
| ENSRNOG00000054204 | Gria2 | 2.048589762 | ENSRNOG00000032798 | Slco3a1 | 1.607604871 |
| ENSRNOG00000004781 | Crmp1 | 2.045110529 | ENSRNOG00000018454 | Apoe | 1.605550902 |
| ENSRNOG00000022710 | Prrg4 | 2.044831258 | ENSRNOG00000019542 | LOC100910979 | 1.604929151 |
| ENSRNOG00000003178 | Mageh1 | 2.040669508 | ENSRNOG00000004208 | Crim1 | 1.604784496 |
| ENSRNOG00000002381 | Bmp3 | 2.030830725 | ENSRNOG00000008738 | Tp53i11 | 1.601326591 |
| ENSRNOG00000020743 | Cyp2s1 | 2.026370643 | ENSRNOG00000012663 | Mfsd6 | 1.598845341 |
| ENSRNOG00000036677 | Slc16a3 | 2.015985047 | ENSRNOG00000042556 | Bmyc | 1.598378197 |
| ENSRNOG00000054391 | Snurf | 2.006982258 | ENSRNOG00000012475 | Prr5 | 1.597738491 |
| ENSRNOG00000001825 | - | 1.999520636 | ENSRNOG00000061147 | Fam189a2 | 1.597665435 |
| ENSRNOG00000019549 | Akap12 | 1.997797947 | ENSRNOG00000016103 | Nkd2 | 1.597133611 |
| ENSRNOG00000046546 | LOC103689954 | 1.993247229 | ENSRNOG00000018338 | Vwa1 | 1.597060192 |
| ENSRNOG00000022595 | LOC100362965 | 1.992643721 | ENSRNOG00000026060 | Arsi | 1.594933664 |
| ENSRNOG00000010457 | Vash1 | 1.99086939 | ENSRNOG00000001057 | Ctxn1 | 1.593439741 |
| ENSRNOG00000059016 | Tspan12 | 1.988850626 | ENSRNOG00000009694 | Bmp4 | 1.587999775 |
| ENSRNOG00000029148 | Pdgfd | 1.98088184 | ENSRNOG00000003003 | Tbc1d19 | 1.586467584 |
| ENSRNOG00000008057 | Krt7 | 1.971336816 | ENSRNOG00000057710 | Ccnd2 | 1.584890951 |
| ENSRNOG00000007377 | Slit3 | 1.969712446 | ENSRNOG00000007660 | Fntb | 1.584414326 |
| ENSRNOG00000002802 | Cxcl1 | 1.956358654 | ENSRNOG00000009835 | Tram1l1 | 1.584372133 |
| ENSRNOG00000009085 | Prkag2 | 1.949771518 | ENSRNOG00000043193 | Smim1 | 1.582462209 |
| ENSRNOG00000006631 | Sema3e | 1.947652487 | ENSRNOG00000020636 | Spint2 | 1.58159273 |
| ENSRNOG00000050152 | Chsy3 | 1.941357824 | ENSRNOG00000021671 | Ldoc1l | 1.577162425 |
| ENSRNOG00000011921 | Dusp4 | 1.934077716 | ENSRNOG00000029134 | Adgrl1 | 1.576173606 |
| ENSRNOG00000025625 | Rnase4 | 1.930302332 | ENSRNOG00000000034 | Nuak2 | 1.575213506 |
| ENSRNOG00000020030 | Crlf1 | 1.929413605 | ENSRNOG00000005046 | Tspan13 | 1.574491563 |
| ENSRNOG00000055765 | LOC103694863 | 1.922039599 | ENSRNOG00000055157 | Pard6g | 1.572874466 |
| ENSRNOG00000017164 | Afap1l2 | 1.920590292 | ENSRNOG00000045843 | Rusc2 | 1.572562296 |
| ENSRNOG00000023720 | Ntm | 1.920466406 | ENSRNOG00000016182 | Tgfa | 1.572521619 |
| ENSRNOG00000059705 | Elmo1 | 1.906763823 | ENSRNOG00000010635 | Igfbp4 | 1.565319494 |
| ENSRNOG00000017869 | Irf8 | 1.898534725 | ENSRNOG00000061910 | Igfbp3 | 1.565264935 |
| ENSRNOG00000054008 | Scamp5 | 1.892918427 | ENSRNOG00000021318 | Epas1 | 1.564653698 |
| ENSRNOG00000014320 | Inhba | 1.891552053 | ENSRNOG00000005041 | Crip2 | 1.563292601 |
| ENSRNOG00000015257 | Coro2b | 1.885478987 | ENSRNOG00000013391 | Sorbs2 | 1.562787625 |
| ENSRNOG00000014548 | Nedd9 | 1.881429601 | ENSRNOG00000024454 | Ccdc149 | 1.561882786 |
| ENSRNOG00000012181 | Lpl | 1.875704717 | ENSRNOG00000000777 | RT1-S3 | 1.560931945 |
| ENSRNOG00000000412 | Slc35f1 | 1.873248153 | ENSRNOG00000013468 | Fam213b | 1.558575039 |
| ENSRNOG00000016021 | Lims2 | 1.872610089 | ENSRNOG00000011634 | Xkr6 | 1.557638539 |
| ENSRNOG00000005772 | Hacd4 | 1.867968839 | ENSRNOG00000013248 | Wwc2 | 1.556369149 |
| ENSRNOG00000012660 | Postn | 1.866156687 | ENSRNOG00000004874 | Flrt3 | 1.555418307 |
| ENSRNOG00000011800 | F3 | 1.864030194 | ENSRNOG00000002215 | Mylk | 1.554022485 |
| ENSRNOG00000033772 | Serpinb9 | 1.863696494 | ENSRNOG00000031675 | Panx3 | 1.553965235 |
| ENSRNOG00000027468 | Slc6a15 | 1.855358674 | ENSRNOG00000056246 | Gls | 1.55161047 |
| ENSRNOG00000043329 | Rnf180 | 1.854481372 | ENSRNOG00000058696 | AABR07013701.1 | 1.550734802 |
| ENSRNOG00000010748 | Mtus1 | 1.85406354 | ENSRNOG00000016322 | Camk2n1 | 1.548160066 |
| ENSRNOG00000001227 | Adarb1 | 1.852791091 | ENSRNOG00000018646 | Hbegf | 1.546954223 |
| ENSRNOG00000007159 | Ccl2 | 1.849762817 | ENSRNOG00000051860 | AC114343.1 | 1.546920397 |
| ENSRNOG00000003869 | Sod3 | 1.847543859 | ENSRNOG00000002810 | Gfpt2 | 1.546056375 |
| ENSRNOG00000022686 | Zdhhc2 | 1.843619147 | ENSRNOG00000013048 | Pde7a | 1.54464772 |
| ENSRNOG00000024602 | Plekha7 | 1.842825393 | ENSRNOG00000017466 | Kif5b | 1.543409988 |
| ENSRNOG00000060603 | Nhsl1 | 1.841764503 | ENSRNOG00000013994 | Enpp1 | 1.542393552 |
| ENSRNOG00000017073 | Car9 | 1.839962451 | ENSRNOG00000002525 | Ptgs2 | 1.542322415 |
| ENSRNOG00000042821 | Cd59 | 1.836203703 | ENSRNOG00000018184 | Tpm1 | 1.541386515 |
| ENSRNOG00000024207 | Fgfrl1 | 1.835099205 | ENSRNOG00000005690 | Lmcd1 | 1.540648593 |
| ENSRNOG00000008137 | Cdk18 | 1.834410115 | ENSRNOG00000030018 | Gzmc | 1.540444118 |
| ENSRNOG00000016758 | Loxl2 | 1.828455324 | ENSRNOG00000045641 | LOC691695 | 1.540391122 |
| ENSRNOG00000005371 | Klhl29 | 1.826026273 | ENSRNOG00000004461 | NEWGENE_6497122 | 1.539209012 |
| ENSRNOG00000002343 | Uchl1 | 1.821410278 | ENSRNOG00000004345 | Daam1 | 1.536140847 |
| ENSRNOG00000011407 | Prag1 | 1.812845441 | ENSRNOG00000004019 | Phlda1 | 1.535185569 |
| ENSRNOG00000009951 | Aif1l | 1.812522443 | ENSRNOG00000011016 | Slc7a2 | 1.535102567 |
| ENSRNOG00000007687 | Sema7a | 1.812311872 | ENSRNOG00000007338 | Fbln2 | 1.534855949 |
| ENSRNOG00000007345 | Amot | 1.810440269 | ENSRNOG00000003759 | Galc | 1.531128136 |
| ENSRNOG00000012674 | Adrb3 | 1.807754244 | ENSRNOG00000018217 | Syt5 | 1.530966335 |
| ENSRNOG00000058039 | Acta2 | 1.806953954 | ENSRNOG00000021669 | Mybl1 | 1.530029667 |
| ENSRNOG00000001156 | Msi1 | 1.806680957 | ENSRNOG00000008754 | Flvcr2 | 1.528841564 |
| ENSRNOG00000019598 | Vegfa | 1.804641435 | ENSRNOG00000001785 | Etv5 | 1.528240123 |
| ENSRNOG00000014776 | Adcy7 | 1.803291872 | ENSRNOG00000009008 | Rab39a | 1.528145147 |
| ENSRNOG00000003772 | Csrp2 | 1.797182398 | ENSRNOG00000021553 | Nckap5 | 1.527843268 |
| ENSRNOG00000013851 | Spry4 | 1.795717047 | ENSRNOG00000023348 | Tbc1d2 | 1.52769172 |
| ENSRNOG00000033527 | Pappa | 1.792936694 | ENSRNOG00000005326 | Cnrip1 | 1.526014335 |
| ENSRNOG00000023473 | Rnf122 | 1.790797774 | ENSRNOG00000018484 | Plk3 | 1.525107808 |
| ENSRNOG00000023257 | Adamts9 | 1.788227236 | ENSRNOG00000037339 | Siglec10 | 1.5249541 |
| ENSRNOG00000024503 | Nbas | 1.786058147 | ENSRNOG00000045948 | Arl10 | 1.518497262 |
| ENSRNOG00000053560 | Rhou | 1.785813124 | ENSRNOG00000006410 | Akap5 | 1.518491997 |
| ENSRNOG00000018322 | Picalm | 1.783771411 | ENSRNOG00000054314 | Kcng1 | 1.517642136 |
| ENSRNOG00000007370 | Rnf144a | 1.78257711 | ENSRNOG00000017132 | Snx30 | 1.517150296 |
| ENSRNOG00000010283 | Cd28 | 1.781657243 | ENSRNOG00000021110 | Mllt11 | 1.516823753 |
| ENSRNOG00000018681 | Nes | 1.781277688 | ENSRNOG00000004839 | B4galnt1 | 1.516766712 |
| ENSRNOG00000016695 | Mmp2 | 1.780885156 | ENSRNOG00000002746 | Fstl1 | 1.51311743 |
| ENSRNOG00000018233 | Gas6 | 1.777012189 | ENSRNOG00000015602 | Cdh2 | 1.511257189 |
| ENSRNOG00000023753 | Afdn | 1.770417167 | ENSRNOG00000002052 | Ccdc80 | 1.510651517 |
| ENSRNOG00000013194 | Rps6ka2 | 1.76670347 | ENSRNOG00000008810 | Nsmf | 1.508983736 |
| ENSRNOG00000000368 | Grik2 | 1.763164025 | ENSRNOG00000012886 | Maff | 1.508858078 |
| ENSRNOG00000013169 | Traf4 | 1.761754738 | ENSRNOG00000025184 | Prss35 | 1.50620447 |
| ENSRNOG00000018992 | Dpysl3 | 1.759184775 | ENSRNOG00000029510 | Plxnb1 | 1.505331367 |
| ENSRNOG00000012095 | Pkia | 1.756323308 | ENSRNOG00000033110 | Svep1 | 1.50522872 |
| ENSRNOG00000003496 | Tbc1d9 | 1.755473916 | ENSRNOG00000007030 | Epha7 | 1.501659859 |
| ENSRNOG00000049484 | Atp9a | 1.745539917 | ENSRNOG00000038539 | Lrrc15 | 1.501047862 |
| ENSRNOG00000007254 | Ttc9 | 1.745470807 | ENSRNOG00000001367 | Gpc2 | 1.500369562 |
| ENSRNOG00000002579 | Parm1 | 1.737660733 |  |  |  |
